# Age-period-cohort analysis of incidence, mortality and disability-adjusted life years of esophageal cancer in global, regional and national regions from 1990 to 2019

**DOI:** 10.1186/s12889-024-17706-8

**Published:** 2024-01-17

**Authors:** Huiying Li, Xianzhi Yang, Aiqi Zhang, Guanying Liang, Yue Sun, Jian Zhang

**Affiliations:** 1 Department of Pathology, Harbin Medical University Cancer Hospital, 150 Haping Road, Harbin City, Heilongjiang Province People’s Republic of China; 2 Emergency Internal Medicine, The First Affiliated Hospital of Harbin Medical University, Harbin City, Heilongjiang Province People’s Republic of China; 3 Academic Department of Science and Technology, Harbin Medical University Cancer Hospital, Harbin City, Heilongjiang Province People’s Republic of China; 4 Department of Thoracic Surgery, Harbin Medical University Cancer Hospital, Harbin City, Heilongjiang Province People’s Republic of China

**Keywords:** Esophageal cancer, Incidence rate, Mortality rate, Disability adjusted life year, Age-period-cohort model

## Abstract

**Objective:**

In view of the high incidence and mortality of esophageal cancer, the latest statistical data on the disease burden of esophageal cancer can provide strategies for cancer screening, early detection and treatment, and help to rationally allocate health resources. This study provides an analysis of the global disease burden and risk factors of esophageal cancer from 1990 to 2019.

**Methods:**

Using the 2019 Global Burden of Disease, Injury and Risk Factor (GBD) data, we present the incidence, mortality and disability-adjusted life years (DALY) of esophageal cancer in 21 regions and 204 countries and different sociodemographic index (SDI) regions from 1990 to 2019. The age-period-cohort model was used to estimate the age, period, and cohort trend of esophageal cancer in different SDI regions. The estimated proportion of DALY attributable to each risk factor from 1990 to 2019.

**Results:**

From 1990 to 2019, the number of new cases of esophageal cancer, the number of deaths and DALY increased by 67.07%, 55.97% and 42.13%, respectively, but age standardized incidence rate (ASIR), age standardized mortality rate (ASMR) and age standardized DALY rate (ASDR) decreased by 19.28%, 25.32% and 88.22%, respectively. Overall, the results of the age-period-cohort model showed that the incidence, mortality, and DALY rates in countries and regions with higher SDI levels showed a downward trend over time and with the passage of time. Conversely, there were no significant changes in incidence and mortality in countries and regions with low SDI levels. In the past 30 years, the incidence and death of esophageal cancer in the world has gradually changed to people over 80 years old, but the population aged 60–79 still accounts for the largest proportion. The global DALY in esophageal cancer is mainly attributable to smoking, followed by alcohol consumption and occupational exposure.

**Conclusions:**

Although ASIR, ASMR and ASDR have decreased significantly, esophageal cancer is still the main factor causing the disease burden worldwide. Public health administrators in low SDI and low-middle SDI countries are high-risk areas for esophageal cancer, and preventive control measures should be implemented to raise awareness, screening, and treatment of esophageal cancer in these areas. Tobacco and alcohol control and reduction of occupational hazards are key steps in reducing the burden of esophageal cancer.

**Supplementary Information:**

The online version contains supplementary material available at 10.1186/s12889-024-17706-8.

## Introduction

Esophageal cancer is one of the deadliest cancers in the world with poor prognosis and high mortality. In 2020, the incidence of esophageal cancer ranks seventh and the overall mortality ranks sixth [1, 2]. Although the global disease burden of esophageal cancer has shown a downward trend in recent years, there are still significant differences in different countries and regions, and the risk of esophageal cancer incidence and mortality is still higher in low sociodemographic index (SDI) and low-middle SDI regions. Esophageal cancer is divided into squamous cell carcinoma and adenocarcinoma [3], squamous cell carcinoma has a high incidence in developing countries [4, 5], and the incidence of adenocarcinoma has increased dramatically in developed countries [6], smoking and excessive alcohol consumption are the main risk factors, followed by social, economic and dietary excess [7].

Age, period, and cohort are the three main intrinsic factors in cancer development, but there is an absolute linear relationship between them, and the age-period-cohort model enhances our understanding of trends in incidence, mortality, and disability-adjusted life years (DALY) rates when adjusting for age, period, and birth cohorts. In this study, we analyzed the global disease burden of esophageal cancer of different sexes from 1990 to 2019. At the same time, the age-standardized incidence, age-standardized mortality rate and age-standardized DALY rate were analyzed in different SDI regions. We hope that through the comprehensive evaluation of esophageal cancer in different regions, we can provide strong data support for policy formulation and balanced allocation of health resources.

## Methods

### Data sources

The 2019 Global Burden of Disease, Injury and Risk Factor (GBD) database provides a powerful resource for understanding the health challenges faced by people all over the World in the 21st century. By tracking the progress within and between countries, GBD contains more comprehensive information and provides data support for medical personnel in disease treatment and prevention. The database estimates the disease burden caused by 369 diseases and injuries in 204 countries and regions by gender, age, year (1990–2019) and location. GBD database uses Bayesian meta-regression modeling tool DisMod-MR 2.1 to estimate the incidence, prevalence, remission rate, excess mortality rate and specific cause mortality rate at the same time, and is revised on the basis of simulation study [8, 9]. The advantage of GBD method is that it applies a consistent method to critically evaluate the available information of each condition, making this information comparable and systematic; results of countries with incomplete estimated data; and report the disease burden with standardized indicators [10]. Age standardized incidence rate (ASIR), age standardized mortality rate (ASMR) and age standardized DALY rate (ASDR) of esophageal cancer are also obtained by gender, location, age, year (from 1990 to 2019) and SDI, and its 95% uncertainty interval (UI) is reported. The website cited in this article was last visited on September 1, 2023.SDI divides the country into five regions (high SDI, high-middle SDI, middle SDI, middle-low SDI and low SDI) according to national per capita income, average years of education and total fertility rate of people over 15 years old. This index ranges from 0 to 1, and the higher the value, the higher the economic level of the region [11].

### Statistical analysis

#### Descriptive analysis

In this study, the change percentage of ASIR, ASMR and ASDR of esophageal cancer during 1990–2019 was calculated, and the change rate = (age-standardized rate in 2019 - age-standardized rate in 1990)/age-standardized rate in 1990 × 100%, and estimates of the 95% UI for 1990 and 2019 were provided for 21 regions worldwide.

#### Age-period-cohort model analysis

Age-period-cohort model analyzes on three dimensions: age, period, and birth cohort. The age effect refers to the differences in disease burden among different age groups caused by factors such as accumulation of social experience and changes in social roles. The period effect reflects the changes in the impact of social, economic, cultural, and demographic factors on all age groups over time, The birth cohort effect represents the change in the impact of the time difference experienced during the initial event on all groups [12].As the relationship among age, period, and cohort is perfectly linear, it is statistically impossible to estimate their independent effects, which is known as the identification problem [13]. We circumvented this issue by producing estimable APC parameters and functions without imposing arbitrary constraints on model parameters [14]. Typically, the age-period-cohort model can be represented as follows:

$$Y\, = \,log{\rm{ }}\left( {abc} \right) = {\rm{ }}\mu \, + \,{\alpha _a} + {\rm{ }}{\beta _b} + {\rm{ }}{\gamma _c} + \varepsilon$$

Where, log (abc) is the natural logarithm of incidence /mortality/DALY rate; µ is the intercept term, representing the disease risk reference level for age, period, and cohort parameters; α_a_ is the age effect of the age group a, a = 1,2,…; β_b_ represents the period effect of the b-th period, b = 1,2,…;γ_c_ represents the queue effect of the c-th queue, c = b-a + n, n represents the number of age groups; ε represents the error term.

In the age-period-cohort model, we divide the data into 16 consecutive age groups, with each age group being 5 years old, ranging from “20–24” years old to “≥ 95” years old; The period is divided into 6 groups every 5 years, from “1990 1994” to “2015 2019”, with a reference period of “2000 2004”; Similarly, the birth cohort is divided into 21 groups every 5 years, from “1895–1899” to “1995–1999”, with a reference period of “1945 1949”. This study used the age-period-cohort web page analysis tool (https://analysistools.cancer.gov/apc/) [15]. This network tool is supported by built-in estimable function algorithms and corresponding Wald tests. The output results of the age-period-cohort model include the longitudinal age curve, which represents the age specific rate of the control cohort adjusted for period bias, representing the impact of age effect on the trend of esophageal cancer changes. The relative risk (RR) of period and birth cohort is the age specific rate ratio of period and cohort based on the selected control period and cohort, representing the impact of period and cohort effects on the trend of esophageal cancer changes. Net drift represents the annual percentage change after considering periods and queues. Local drift represents the annual percentage change in logarithms of different age groups and birth cohorts. Drift above 0.0% per year is considered an increasing trend, while drift below 0.0% per year shows a decreasing percentage change next year. Two-sided statistical test, *P* < 0.05 is statistically significant [12].

#### Risk factor analysis

In addition, we calculated the changing trend of DALY proportion of esophageal cancer caused by smoking, drinking, high BMI, chewing tobacco and dietary risks from 1990 to 2019. The GBD 2019 estimation of attributable burden followed the general framework established for comparative risk assessment (CRA) used in GBD since 2002 [16]. CRA can be divided into six key steps: inclusion of risk–outcome pairs in the analysis; estimation of relative risk as a function of exposure; estimation of exposure levels and distributions; determination of the counterfactual level of exposure; computation of population attributable fractions and attributable burden; and estimation of mediation of different risk factors through other risk factors, to compute the burden attributable to various combinations of risk factors [17].

## Results

### The burden of esophageal cancer at global and regional level

**Fig. 1:**
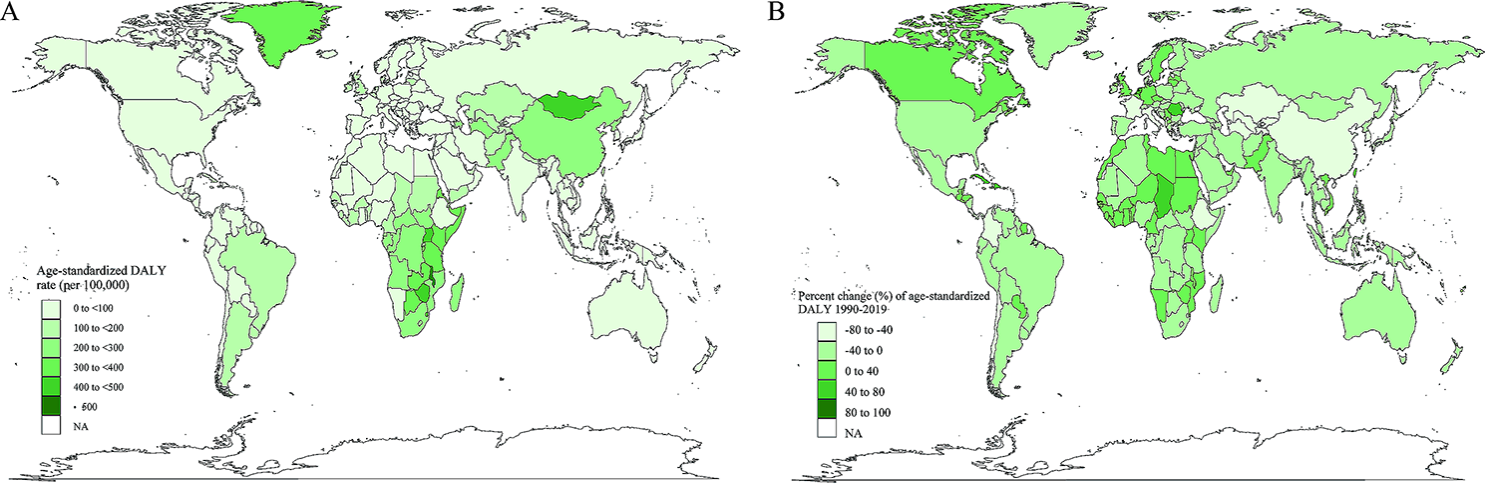
(A)Geographical distribution of ASDR of esophageal cancer in 2019. (B)The percentage change in ASDR of esophageal cancer for 204 countries and territories from 1990 to 2019

**Fig. 2:**
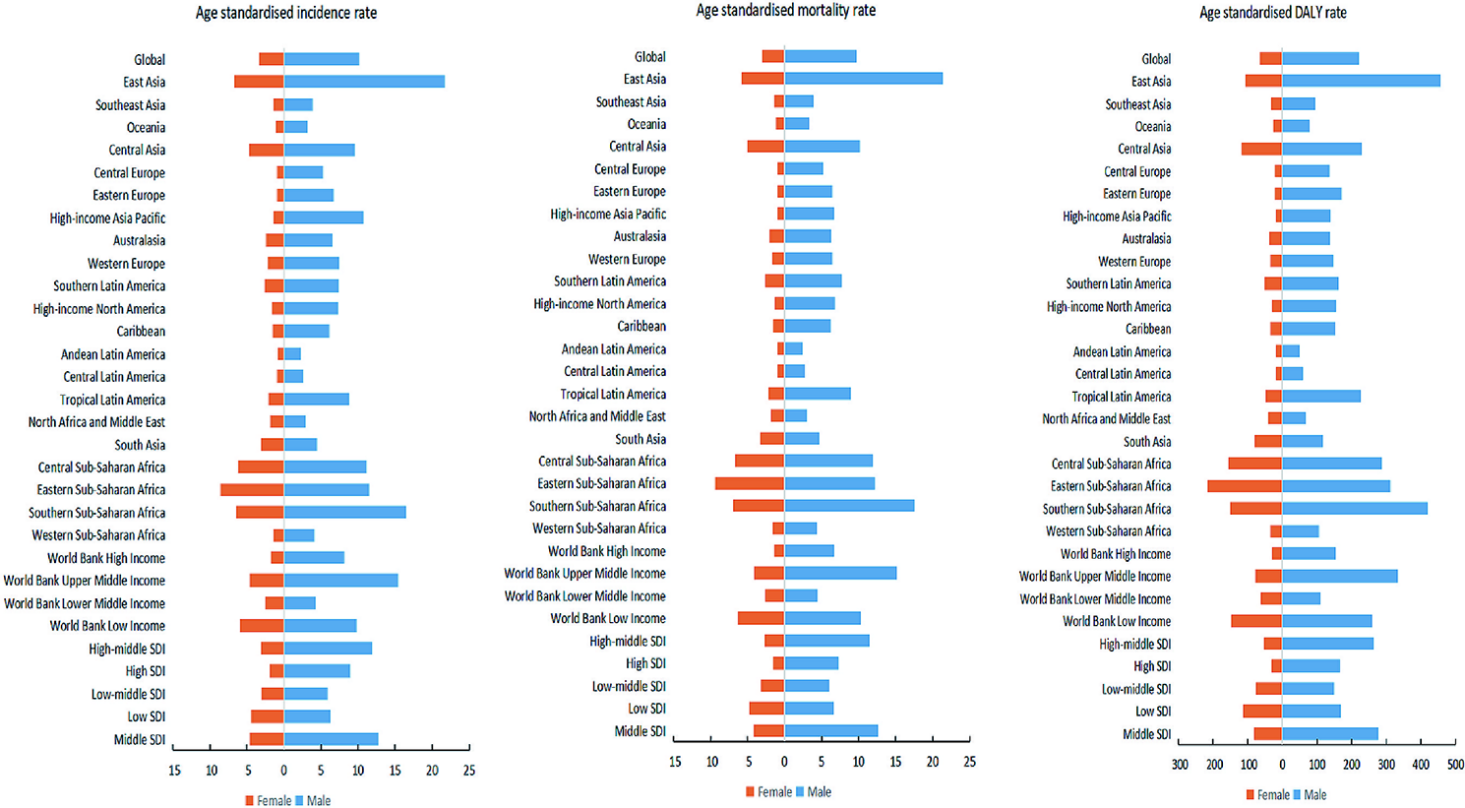
The age-standardized esophageal cancer incidence, mortality, and DALY rates of 31 regional in 2019 by gender

On a global scale, the number of new cases of esophageal cancer increased from 319,969 (253,395 − 351,210) in 1990 to 534,563 (466,513–595,342) in 2019, From 1990 to 2019, the number of deaths of esophageal cancer increased from 319,332 (248,666 − 350,802) to 498,067 (438,411–551,462), and DALY increased from 8,208,267 (6,334,289-9,075,711) to 11,666,017 (10,378,747 − 12,938,949). From 1990 to 2019, ASIR of worldwide esophageal cancer decreased from 8.06 (6.41–8.83) per 100,000 to 6.51 (5.69–7.25) per 100,000, a decrease of 19.28%; ASMR decreased from 8.18 (6.40–8.97) per 100,000 to 6.11 (5.38–6.76) per 100,000, a decrease of 25.32%; ASDR decreased from 199.28 (154.25-219.99) per 100,000 to 33.43 (26.85–41.96) per 100,000, a decrease of 88.22%. However, this trend was not uniform across the globe. Only high-income regions in North America and Western sub-Saharan Africa experienced an increase in both ASIR and ASMR. From 1990 to 2019, Central Asia recorded the most significant decline in ASIR and ASMR, both decreasing by over 51%. In contrast, Southeast Asia and Latin America showed substantial increases in ASDR, rising by 267.75% and 212.98%, respectively (Table 1). At the country level, in 2019, nations in sub-Saharan Africa (such as Uganda, Malawi, Zambia, Zimbabwe), Central Asia (such as Mongolia), East Asia (such as China), and North America (such as Greenland) reported the highest ASIR, ASMR and ASDR. The Northern Mariana Islands saw the largest increase in these age-standardized metrics during the study period (Fig. 1, Figure S1, Figure S2). Additionally, across the 21 World regions, the incidence, mortality, and DALY rates of esophageal cancer in men were more than threefold higher than in women (Fig. 2).

**Table 1:** Incidence number, Deaths, and DALY for esophageal cancer in 1990 and 2019 and percentage change of age standardized rates by location

Location	1990						2019						Percentage change 1990 to 2019		
	Incidence number	ASIR (95% UI)	Death Number	ASMR (95% UI)	DALYs (95% UI)	ASDR (95% UI)	Incidence number	ASIR (95% UI)	Death Number	ASMR (95% UI)	DALY(95% UI)	ASDR (95% UI)	ASIR (95% UI)	ASMR (95% UI)	ASDR (95% UI)
Global	319,969 (351,210–253,395)	8.06(8.83–6.41)	319,332 (350,802 − 248,666)	8.18(8.97–6.4)	8,208,267 (9,075,711 -6,334,289)	199.28(219.99-154.25)	534,563 (595,342–466,513)	6.51(7.25–5.69)	498,067 (551,462 − 438,411)	6.11(6.76–5.38)	11,666,017 (12,938,949 − 10,378,747)	33.43(41.96–26.85)	-19.28(-17.94–11.37)	-25.32(-24.61–15.91)	-83.22(-80.93–82.59)
East Asia	176,236(205,921 − 114,354)	20.48(23.74–13.39)	179,088 (208,583 − 114,836)	21.55(24.88–13.89)	4,562,936 (5,350,134 -2,892,718)	495.62(579.37-316.22)	284,908 (338,886 − 220,166)	13.72(16.25–10.64)	263,307 (314,860 − 209,014)	12.96(15.37–10.19)	5,922,865 (7,156,234 -4,733,467)	83.49(95.11–72.58)	-33(-31.53–20.58)	-39.88(-38.22–26.64)	-83.15(-83.58–77.05)
Southeast Asia	7,090 (8,059 − 6,040)	2.75(3.11–2.37)	7,181 (8,103 -6,113)	2.89(3.25–2.48)	201,111 (228,343 − 169,876)	71.63(80.94–60.71)	15,543 (18,202 − 13,193)	2.54(2.97–2.18)	15,330 (17,964 − 13,164)	2.59(3.05–2.24)	403,725 (472,284–342,843)	263.43(332.94-200.49)	-7.5(-4.59–7.76)	-10.35(-6.15–9.75)	267.75(311.33-230.25)
Oceania	63 (89 − 47)	2.18(3.06–1.69)	63 (89 − 48)	2.34(3.26–1.82)	1,828 (2,558 -1,354)	55.29(77.63–41.88)	147 (196 − 110)	2.15(2.89–1.66)	147 (197 − 111)	2.3(3.05–1.78)	4,213 (5,650 -3,133)	139.79(154.98-124.44)	-1.42(-5.62–1.75)	-1.6(-6.59–2.08)	152.86(99.64-197.17)
Central Asia	6,415 (6,653 -6,206)	13.8(14.34–13.32)	6,622 (6,871 -6,392)	14.53(15.12-14)	170,813 (177,210 − 165,262)	351.73(365.68-340.36)	4,834 (5,679 -4,274)	6.7(7.75–5.95)	4,924 (5,769 -4,359)	7.08(8.15–6.32)	129,818 (153,552 − 114,167)	88.3(91.37–84.99)	-51.48(-45.93–55.31)	-51.27(-46.08–54.89)	-74.9(-75.01–75.03)
Central Europe	4,277 (4,386 -4,165)	2.9(2.97–2.81)	4,328 (4,443 -4,206)	2.96(3.03–2.86)	115,388 (118,310 − 112,612)	78.12(80.02–76.23)	5,853 (6,664 -5,109)	2.89(3.3–2.52)	5,856 (6,670 -5,124)	2.86(3.25–2.49)	143,701 (164,319 − 124,717)	67.83(81.61–51.54)	-0.16(11.13–10.44)	-3.46(7.12–12.94)	-13.17(1.99–32.39)
Eastern Europe	12,165 (12,875 − 11,699)	4.27(4.53–4.1)	12,145 (12,854 − 11,682)	4.29(4.56–4.13)	320,906 (341,033–308,770)	112.18(119.59-107.77)	11,086 (12,604 -9,669)	3.25(3.69–2.83)	10,655 (12,077 − 9,298)	3.1(3.51–2.71)	277,541 (316,272 − 240,922)	275.44(331.75-221.92)	-23.92(-18.49–31.07)	-27.74(-22.96–34.27)	145.53(177.4-105.92)
High-income Asia Pacific	13,198 (13,571 − 12,650)	6.5(6.69–6.23)	10,133 (10,392 -9,655)	5.06(5.2–4.83)	243,863 (250,246 − 229,035)	117.55(120.64-110.61)	25,159 (29,616 − 21,213)	5.71(6.76–4.83)	16,337 (17,795 − 14,650)	3.53(3.84–3.22)	306,118 (333,763 − 281,921)	131.03(137.7-124.29)	-12.19(0.93–22.5)	-30.39(-26.08–33.32)	11.47(14.14–12.37)
Australasia	1,076 (1,120 -1,024)	4.58(4.77–4.36)	1,016 (1,054–970)	4.33(4.5–4.12)	22,212 (23,001–21,393)	95.48(98.89–91.9)	2,192 (2,707 -1,767)	4.41(5.46–3.55)	2,035 (2,218 -1,830)	4.02(4.39–3.65)	39,885 (43,385 − 36,426)	85.18(92.42–78.02)	-3.72(14.53–18.66)	-7.24(-2.44–11.52)	-10.78(-6.54–15.11)
Western Europe	26,996 (27,496 − 26,237)	4.84(4.92–4.71)	25,954 (26,463 − 25,117)	4.59(4.68–4.45)	601,312 (611,699 − 588,465)	112.47(114.44-110.22)	40,174 (45,706 − 35,133)	4.64(5.29–4.06)	34,847 (36,620 − 32,416)	3.87(4.06–3.64)	706,817 (741,655–669,630)	90.7(104.79–76.86)	-3.99(7.46–13.71)	-15.7(-13.2–18.12)	-19.35(-8.44–30.27)
Southern Latin America	3,382 (3,495 -3,259)	7.43(7.68–7.15)	3,543 (3,668 -3,408)	7.89(8.18–7.56)	80,442 (83,110 − 77,833)	173.23(179-167.48)	3,945 (4,943 -3,158)	4.7(5.89–3.75)	4,067 (4,359 -3,769)	4.82(5.15–4.47)	83,206 (89,098 − 77,617)	215.22(294-115.53)	-36.7(-23.24–47.53)	-38.96(-37.02–40.86)	24.24(64.24–31.02)
High-income North America	13,204 (13,565 − 12,784)	3.86(3.96–3.75)	12,515 (12,862 − 12,085)	3.62(3.72–3.5)	292,713 (300,085–285,562)	88.99(91.21–86.96)	26,162 (30,594 − 22,461)	4.22(4.96–3.63)	24,152 (25,147 − 22,876)	3.84(3.99–3.65)	524,630 (544,030–503,915)	151.03(174.68-121.17)	9.36(25.03–3.11)	6.05(7.32–4.23)	69.71(91.52–39.34)
Caribbean	985 (1,062–921)	3.83(4.14–3.59)	1,022 (1,106–955)	4.03(4.35–3.75)	24,294 (26,553 − 22,600)	92.36(100.7-86.04)	1,920 (2,200 -1,641)	3.69(4.23–3.15)	1,923 (2,197 -1,649)	3.7(4.23–3.17)	47,316 (54,718 − 40,107)	37.96(44.75–32.14)	-3.7(2.19–12.04)	-8.15(-2.89–15.43)	-58.89(-55.55–62.64)
Andean Latin America	386 (436 − 325)	1.95(2.2–1.64)	412 (464 − 347)	2.14(2.41–1.8)	9,890 (11,215 -8,331)	46.75(53.01–39.45)	827 (1,021–670)	1.51(1.85–1.22)	889 (1,090–723)	1.63(2-1.33)	18,839 (23,638 − 15,081)	88.72(92.32–84.78)	-22.86(-15.79–25.68)	-23.69(-17.07–26.22)	89.79(74.16-114.92)
Central Latin America	1,909 (1,965 -1,838)	2.39(2.47–2.28)	2,008 (2,070 − 1,921)	2.6(2.68–2.46)	48,243 (49,490 − 46,833)	55.97(57.55–54.09)	3,869 (4,511 -3,281)	1.66(1.94–1.41)	4,021 (4,707 -3,391)	1.74(2.04–1.47)	90,775 (107,044–76,781)	175.17(202.51-145.44)	-30.54(-21.55–38.29)	-32.79(-24.1–40.26)	212.98(251.91-168.87)
Tropical Latin America	6,131 (6,371 -5,908)	6.66(6.93–6.39)	6,205 (6,465 -5,949)	6.94(7.26–6.62)	170,087 (176,763 − 164,206)	172.8(179.68-166.64)	12,684 (13,294 − 11,993)	5.17(5.42–4.87)	12,767 (13,448 − 11,996)	5.25(5.53–4.92)	328,430 (345,187–311,722)	200(233.85-165.89)	-22.4(-21.82–23.77)	-24.41(-23.79–25.68)	15.74(30.14–0.46)
North Africa and Middle East	4,371 (5,236 -3,077)	2.54(3.03–1.85)	4,442 (5,290 -3,157)	2.69(3.19–1.97)	122,368 (147,739 − 81,913)	65.39(78.48–45.45)	10,024 (11,436 -7,415)	2.36(2.66–1.79)	9,968 (11,383 -7,385)	2.44(2.75–1.85)	259,488 (301,673 − 183,343)	74.7(85.35–64.49)	-7.21(-12.08–3.39)	-9.2(-13.85–6.02)	14.25(8.75–41.89)
South Asia	25,604 (32,082 − 22,501)	4.52(5.67–3.99)	25,840 (32,260 − 22,630)	4.79(5.94–4.2)	744,462 (927,062–652,926)	117.87(146.78-103.09)	53,488 (72,051 − 46,152)	3.78(5.1–3.27)	54,161 (72,771 − 46,992)	3.93(5.25–3.41)	1,476,590 (1,962,191 -1,282,692)	98.29(131.08–85.53)	-16.48(-10.05–18.04)	-17.95(-11.68–18.95)	-16.61(-10.7–17.03)
Central Sub-Saharan Africa	2,482 (3,296 -1,149)	10.84(14.27–5.04)	2,528 (3,312 -1,184)	11.57(15.02–5.38)	72,458 (94,381 − 33,619)	285.62(372.7-132.66)	4,431 (6,020 − 2,378)	8.41(11.59–4.48)	4,509 (6,141 -2,426)	8.98(12.41–4.79)	127,510 (172,764 − 68,514)	55.58(63.78–40.51)	-22.38(-18.78–11.16)	-22.41(-17.41–10.88)	-80.54(-82.89–69.47)
Eastern Sub-Saharan Africa	8,438 (9,881 -6,393)	11.18(13.03–8.49)	8,621 (10,074 − 6,513)	11.87(13.86-9)	244,963 (288,763 − 182,809)	296.41(348.07-223.81)	16,391 (20,713 − 12,431)	10.03(12.6–7.71)	16,940 (21,344 − 12,941)	10.77(13.48–8.28)	476,744 (608,685 − 361,802)	95.82(100.45–91.39)	-10.3(-3.3–9.18)	-9.26(-2.72–8)	-67.67(-71.14–59.17)
Southern Sub-Saharan Africa	3,724 (4,579 -2,788)	13.31(16.49–9.93)	3,764 (4,657 -2,820)	13.85(17.14–10.36)	106,970 (131,561 − 79,140)	356.58(439.36-265.85)	5,941 (6,943 -5,316)	10.66(12.29–9.56)	6,095 (7,002–5,489)	11.3(12.77–10.23)	159,882 (188,481 − 142,561)	164.79(193.06-145.56)	-19.92(-25.47–3.78)	-18.4(-25.48–1.2)	-53.79(-56.06–45.25)
Western Sub-Saharan Africa	1,838 (2,145 -1,584)	2.1(2.44–1.82)	1,900 (2,234 -1,642)	2.24(2.6–1.94)	51,009 (60,395 − 43,559)	54.31(64.1-46.63)	4,986 (5,992 -3,776)	2.71(3.21–2.06)	5,135 (6,146 -3,916)	2.89(3.42–2.2)	137,923 (167,521 − 104,912)	61.73(72.38–52.74)	28.85(31.6-12.83)	29.32(31.23–13.41)	13.65(12.93–13.1)
High-middle SDI	86,734 (95,038–73,335)	8.08(8.85–6.86)	88,112 (96,912 − 73,501)	8.34(9.14–6.98)	2,235,279 (2,479,332 -1,851,568)	202.97(224.69-168.18)	145,267 (169,316 − 113,247)	7.06(8.22–5.5)	135,757 (156,606 − 108,339)	6.62(7.62–5.29)	3,105,596 (3,596,286 -2,487,365)	75.76(82.63–70.65)	-12.64(-7.05–19.76)	-20.64(-16.54–24.2)	-62.67(-63.22–57.99)
High SDI	52,157 (53,200 − 50,608)	5.07(5.17–4.93)	47,439 (48,389 − 45,967)	4.59(4.68–4.45)	1,116,159 (1,135,517 -1,091,372)	111.26(113.23-108.87)	95,911 (105,092 − 86,719)	5.2(5.7–4.71)	79,088 (83,089 − 73,600)	4.18(4.38–3.93)	1,653,972 (1,731,345 -1,570,861)	111.27(155.69–99.04)	2.45(10.1–4.31)	-8.85(-6.34–11.61)	0.01(37.5–9.04)
Low-middle SDI	30,394 (39,269 − 26,887)	5.07(6.54–4.51)	30,842 (39,603 − 27,388)	5.34(6.86–4.75)	863,275 (1,104,285–763,527)	131.98(169.03-117.04)	59,864 (84,056 − 52,707)	4.37(6.17–3.85)	60,670 (85,565 − 53,987)	4.53(6.39–4.02)	1,611,655 (2,250,321 -1,433,392)	86.65(107-77.42)	-13.83(-5.67–14.62)	-15.2(-6.81–15.35)	-34.35(-36.7–33.85)
Low SDI	14,366 (16,558 − 11,901)	6.02(6.89-5)	14,642 (16,772 − 12,029)	6.38(7.31–5.24)	417,176 (481,147–342,900)	159.59(183.21-131.26)	28,132 (33,412 − 23,219)	5.39(6.4–4.48)	28,684 (34,252 − 23,834)	5.69(6.75–4.75)	805,543 (973,246–662,160)	141.09(168.89-116.53)	-10.52(-7.03–10.49)	-10.86(-7.68–9.27)	-11.59(-7.82–11.22)
Middle SDI	136,240 (157,389 − 81,440)	13.49(15.54–8.11)	138,218 (159,517 − 80,643)	14.13(16.22–8.38)	3,574,428 (4,144,035 − 2,120,546)	330.13(382.91–194.3)	205,237 (237,934 − 164,588)	8.42(9.75–6.7)	193,720 (223,774 − 157,830)	8.15(9.39–6.54)	4,485,644 (5,192,821 -3,737,169)	200.96(244.17–150)	-37.57(-37.31–17.41)	-42.34(-42.09–21.99)	-39.13(-36.23–22.8)

### Influence of socio-demographic index on esophageal cancer incidence, mortality and DALYs

**Fig. 3:**
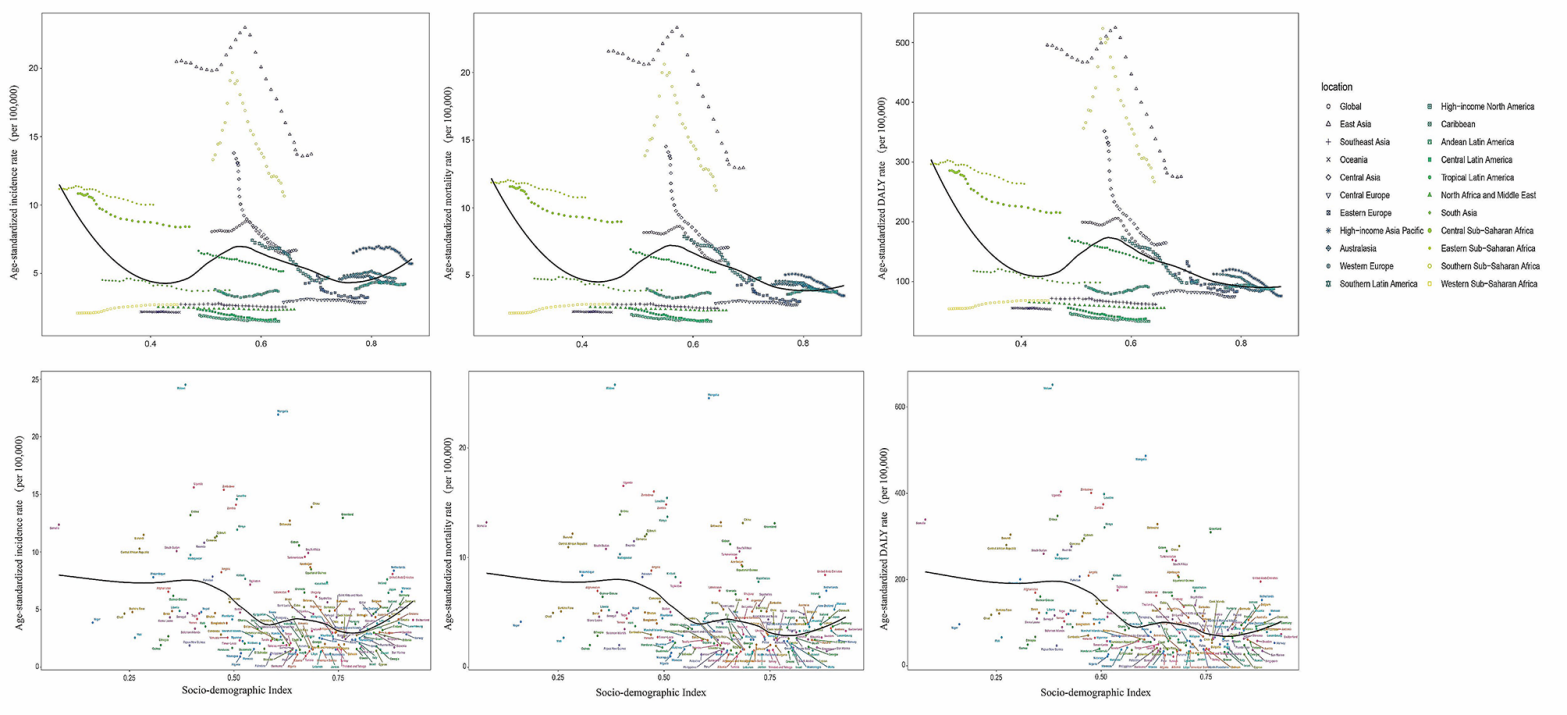
Age-standardized incidence, mortality, and DALY rates for esophageal cancer for 21 GBD regions (A) and 195 countries and territories (B) by Socio-demographic Index, 1990–2019

Overall, countries and regions with high SDI levels usually have low incidence, mortality, and DALY rates. Most countries and regions with low SDI levels have relatively high mortality rates. In countries and regions with middle SDI levels, incidence, mortality, and DALY rates are significantly higher. In terms of gender, with the increase of SDI level in different countries and regions, women’s incidence, mortality, and DALY rates decline more than men’s, especially in low SDI regions and low-middle SDI regions. The distribution and changes of esophageal cancer incidence, mortality, and DALY rates in 21 regions and 204 countries with different SDI levels are shown in Fig. 3, Figure S3, and Figure S4 respectively.

### Time trend of incidence, deaths, and disability adjusted life year distribution of esophageal cancer in different age groups

**Fig. 4:**
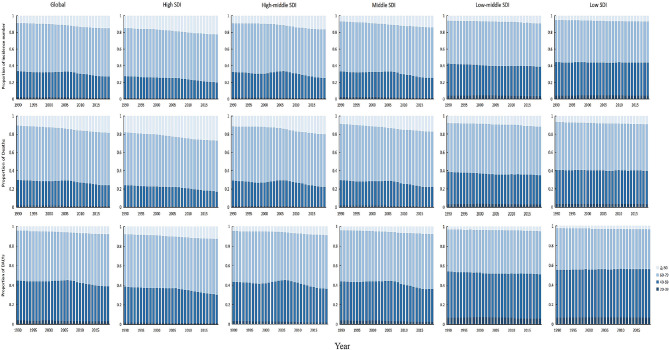
Trends in the age distribution of incidence number, deaths and DALY owing to esophageal cancer across countries and regions with different SDI for the entire population, 1990–2019

Figure 4 shows the time trend of the total number of esophageal cancer cases, deaths, and DALY distribution in different age groups from 1990 to 2019. In the past 30 years, the global number of cases and death of esophageal cancer has gradually shifted towards people over 80 years old, but the proportion of people aged 60 to 79 is still the largest. The number of cases, deaths, and DALY varies among regions with different SDI. In high SDI, middle- high SDI, and middle SDI regions, the proportion of esophageal cancer cases and deaths in the age group over 80 gradually increases. The number of cases and deaths in the age group between 40 and 59 decreases year by year, and by 2019, the number of deaths in the age group over 80 in high SDI regions even exceeded that in the age group between 40 and 59. In low-middle SDI and low SDI regions, there have been changes in the age distribution of the number of cases and deaths of esophageal cancer, with a decrease in the proportion of deaths occurring over the age of 80 and an increase in the proportion of people aged 40–59.

### The impact of age, period, and cohort factors on the incidence, mortality, and disability adjusted life years of esophageal cancer

**Fig. 5:**
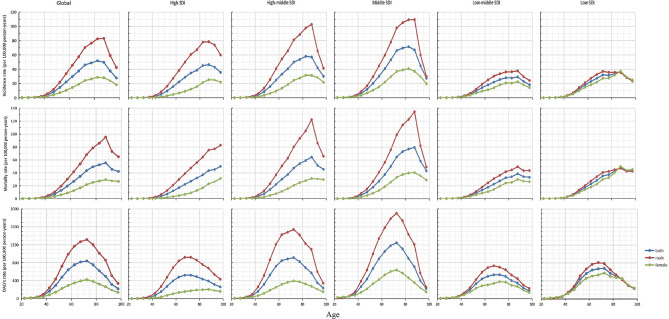
Age effect analysis of esophageal cancer incidence, mortality, and disability adjusted life years globally and in countries and regions with different SDI from 1990 to 2019

This study analyzed the impact of age, period, and cohort effects on the global and different SDI regions. As shown in Fig. 5 and Figure S5, Table S1 for the age-related impact in the World and different SDI regions, it can be clearly seen that except for the high SDI regions, the mortality rate continues to increase with age, the incidence, mortality, and DALY rates in other regions show a trend of first rising and then declining with age. The incidence and mortality rates began to decline after reaching the peak in the 85–90 age group, and the DALY rate reached the peak in the 70–75 age group. Among them, the increase and decrease of male incidence, mortality and DALY rates are greater than that of female with the increase of age. Compared with low-middle SDI and low SDI areas, incidence, mortality, and DALY rates of all age groups in high SDI, high-middle SDI and middle SDI areas are higher.

**Fig. 6:**
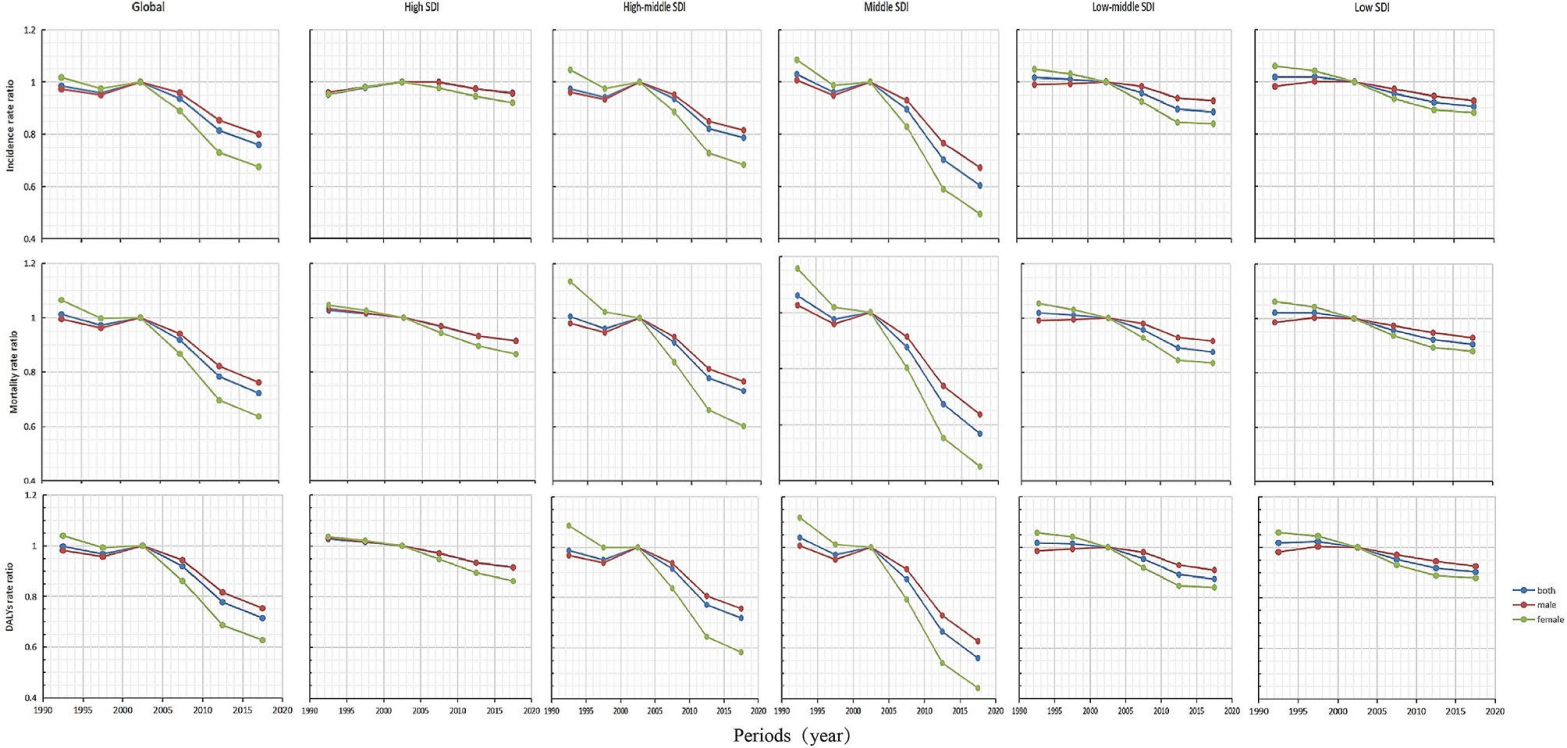
Analysis of the period effects of esophageal cancer incidence, mortality, and DALY globally and in countries and regions with different SDIs from 1990 to 2019

The global and different SDI regions have different period effects. At the global level, the period effect of incidence, mortality and DALY rates gradually decreases over time. In countries and regions with high SDI, the period effect of incidence rate almost remained unchanged from 1990 to 2019, and mortality and DALY rates only showed a slight downward trend. Countries and regions with high -middle SDI and middle SDI experienced significant risk reduction periods after 2002. In contrast, the risk reduction trend in regions with low -middle SDI and low SDI was not significant between 1990 and 2019 and remained basically unchanged after 2012 (Fig. 6, Table S1).

**Fig. 7:**
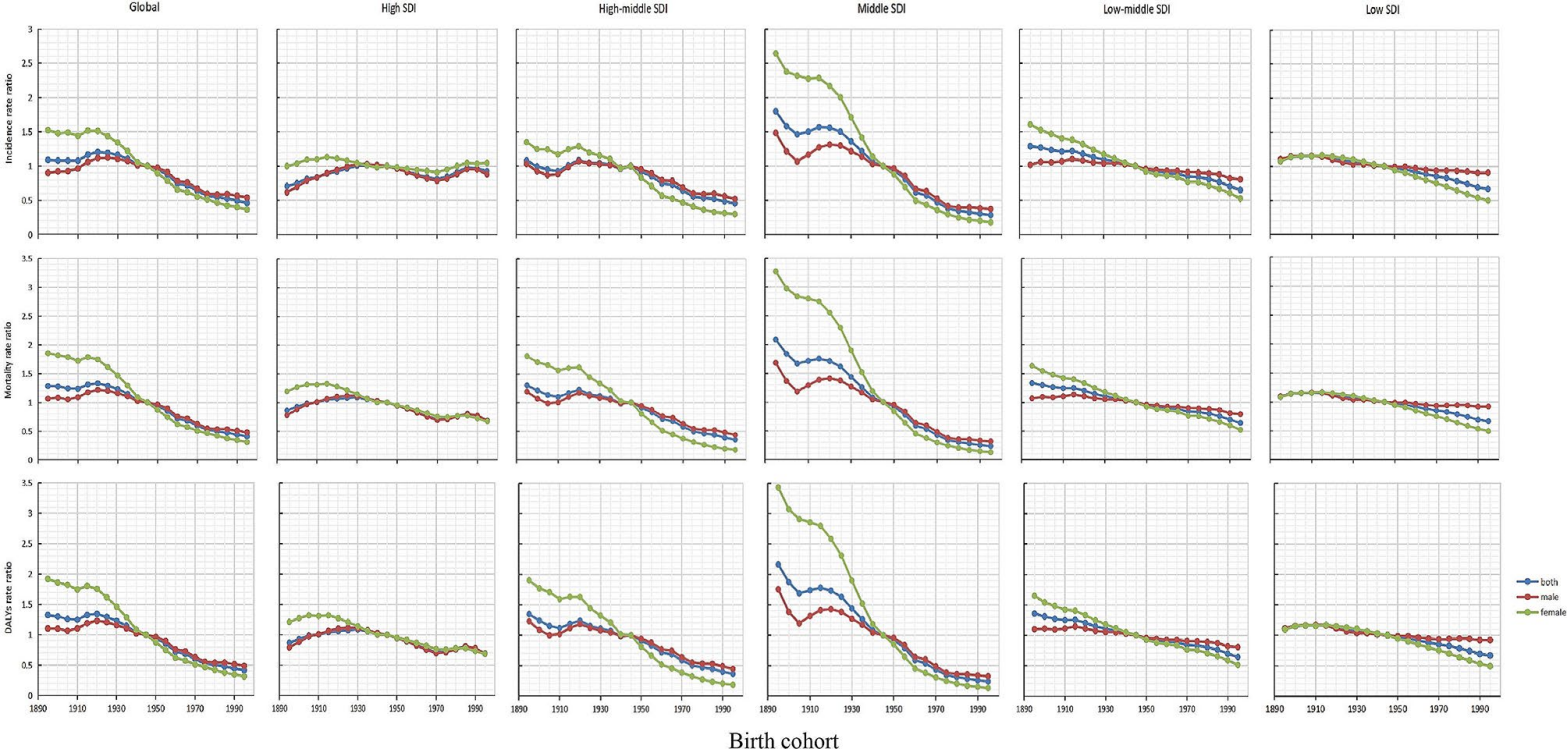
Analysis of the cohort effects of esophageal cancer incidence, mortality, and DALY globally and in countries and regions with different SDIs from 1990 to 2019

Like the period effect, in the global, high-middle SDI and middle SDI regions, with the backward shift of birth year, the risk of birth queue is significantly reduced, especially in middle SDI regions. In contrast, the incidence, mortality, and DALY rates risk in areas with high SDI, low-middle SDI and low SDI have not changed significantly. Except for areas with high SDI, the risk of birth queue in other areas showed a trend that women were higher than men in 1890–1945, and then men were higher than women (Fig. 7, Table S1).

### Analysis of risk factors of esophageal cancer

**Fig. 8:**
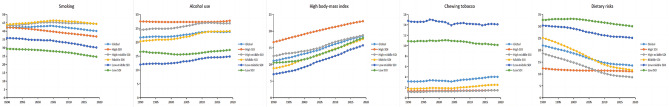
Trends in the proportion of age adjusted life years for esophageal cancer caused by different risk factors in countries and regions with different SDI levels from 1990 to 2019

At the global level, among the risk factors of esophageal cancer from 1990 to 2019, smoking accounted for the highest proportion, followed by alcohol use. With the passage of time, the proportion of high BMI gradually increased, and the proportion of dietary risks gradually decreased. Among the risk factors in high SDI areas, alcohol use and high BMI are higher than those in other areas. Among the risk factors in the middle SDI area, smoking accounts for a relatively high proportion, and the proportion of dietary risks is decreasing year by year. chewing tobacco and dietary risks accounted for the highest proportion in low-middle SDI and low SDI areas, and there was no significant change between 1990 and 2019 (Fig. 8).

## Discussion

### Disease burden of esophageal cancer in different regions

In the past 30 years, the global population has gradually increased, increasing by 44.6% compared to 1990 [18]. In 2019, the absolute number of incidence, death and DALY of esophageal cancer increased compared with 1990, but ASIR and ASMR of esophageal cancer decreased, and the ASDR even decreased by 88.22%.From 1990 to 2019, the observed decline in ASIR and ASMR may be due to the improvement of some social and environmental factors [19]. In addition, it is necessary to focus on high-risk areas such as high-income areas in North America, Western sub-Saharan Africa, Mongolia, and China, and identify the reasons for the high ASIR and ASMR of esophageal cancer. Adenocarcinoma is the main subtype in countries such as the United States, Australia, and Western Europe [20]. Türkiye, Iran, Kazakhstan, and northern China form the “Asian Esophageal Cancer Belt” [21], where esophageal cancer has a high incidence and mortality rates, which may be related to local environmental factors and living habits [22]. There is an “African Esophageal Cancer Corridor” in Africa, which is a high incidence area for esophageal squamous cell carcinoma. It is currently inferred that the occurrence of esophageal cancer in Africa is related to micronutrient deficiency [23]. In the whole population of esophageal cancer, ASIR, ASMR, and ASDR of men are more than three times higher than those of women, which may be related to hormone levels, metabolism and immunity between men and women [24].

### Effect of different SDIs on the disease burden of esophageal cancer

Globally, the burden of esophageal cancer has decreased significantly, but the cases of esophageal cancer has increased in some countries or regions, especially in high SDI areas, which may be related to the increase of population in high SDI areas. Therefore, it is necessary to further strengthen health education for migrant populations and popularize knowledge on disease prevention and control. On the contrary, the mortality rate in high SDI areas is significantly lower than that in other areas, indicating that high-income areas have better medical conditions [25]. Improving medical care is a key factor in preventing and treating cancer incidence and mortality. With the increase of SDI levels, the disease burden of 21 regions and 204 countries worldwide shows a trend of first decreasing, then increasing, and then decreasing, indicating that the disease burden of esophageal cancer is heavier in the middle SDI region, which may be related to the high smoking rate in the middle SDI region [26].Because smoking is the main cause of increased incidence of esophageal cancer. Between 1990 and 2019, the incidence and mortality rates of esophageal cancer gradually increased among people over the age of 80 in the global, high SDI, high- middle SDI, and middle SDI regions, which is closely related to global population aging [27]. In low to middle SDI and low SDI regions, the proportion of people aged 40–59 is higher than other regions and tends to be younger, which is related to local economic, educational, and cultural factors [28]. Due to the low level of economy and education, people do not have enough awareness of cancer and cannot prevent it in time [29].

### Effect of age, period, and cohort effect on esophageal cancer

The results of age-period-cohort model show that in the past 30 years, there are different ages, periods, and birth queues among different sexes in the world and different SDI regions. The age effect suggests that the incidence, mortality, and DALY rates of esophageal cancer gradually increase with the increase of age, reaching the peak at the age of 85–89, which indicates that with the intensification of population aging, the number of elderly patients increases, and they are usually not adequately treated, which will inevitably affect the disease burden [27]. Therefore, there is a need to provide these elderly patients with adequate treatment options, including increased life expectancy, reduced comorbidities, and improved quality of life [30, 31].. The results of period effect show that the risk of incidence, mortality and DALY rates in men is significantly higher than that in women after 2002, suggests that prophylactic screening for men should be increased. From 1990 to 2019, the period effect gradually decreased over time, and the downward trend was the most obvious in the middle SDI area. This may be closely related to the implementation of smoking cessation and alcohol restriction in developing countries. Cohort effect shows that the birth cohort risk of esophageal cancer in different populations born at a specific time point shows a downward trend in incidence, mortality, and DALY rates. The analysis of age-period-cohort model in different SDI regions shows that the period effect and birth cohort effect have the most significant risk reduction trends in high-middle SDI regions and middle SDI regions. The changes of period effect and birth queue effect in low-middle SDI area and low SDI area only decreased slightly. Studies have shown that period and cohort effects have a significant impact on the incidence of esophageal cancer in regions with high SDI, such as Australia and the United States [32]. Therefore, the prevention and control measures of esophageal cancer should be strengthened in low-middle SDI areas and low SDI areas.

### Influence of risk factors for esophageal cancer

In this study, alcohol use, smoking, high BMI, chewing tobacco and dietary risks were included as important risk factors for esophageal cancer. The main factors of esophageal squamous cell carcinoma are excessive alcohol use and smoking, and esophageal adenocarcinoma is mainly secondary to gastroesophageal reflux disease, and the risk of gastroesophageal reflux disease increases with the increase of body weight BMI [33]. Men and women are more likely to die from esophageal cancer than women due to the difference in smoking rates [34, 35]. Therefore, increasing smoking cessation rates is of great significance in preventing the occurrence of esophageal cancer. In the past 30 years, the risk of high BMI has gradually increased, indicating that we should pay attention to weight and reduce the incidence of obesity. In addition, studies have shown that compared to individuals with low genetic predispositions, individuals with high genetic obesity tendencies have a higher risk of developing esophageal cancer, and early screening for obesity should be strengthened [36, 37]. In low-middle SDI areas and low SDI areas, the risk of chewing tobacco and diet is relatively high, which may be related to population and social economy [38]. Because there are obstacles in eliminating risk factors, it is difficult to achieve primary prevention. At present, the basic principle of esophageal cancer prevention is to identify high-risk groups in the early stage of the disease so that they can get timely treatment and strengthen monitoring to achieve secondary prevention [39].

GBD studies presented comprehensive, high-quality estimates of the global burden of disease, but they had some limitations based on data collection procedures, treatments, and individual biases. Data collection procedures and data sources vary across countries and regions. In addition, there may be significant differences in incidence between different regions of the same country, whereas GBD does not have a detailed record of development within the country. In addition to this, another limitation is that, as an age-period-cohort model-based study, we provide long-term trends in esophageal cancer, but the effects of many suspected risk factors have not been well studied.

## Conclusion

Although ASIR, ASMR, and ASDR of esophageal cancer show a downward trend globally, esophageal cancer is still a disease with a heavy burden in the World [5, 40]. In recent years, the overall disease burden of esophageal cancer has gradually decreased with the increase of SDI, but the disease burden in middle SDI areas has significantly increased. Therefore, screening and prevention and control of esophageal cancer in these areas should be strengthened. At the same time, control for known potential risk factors for esophageal cancer, such as reducing smoking and alcohol consumption, controlling body fat, and paying attention to diet. If possible, collecting disease burden and related genetic information of esophageal cancer among different races can provide strong evidence for global esophageal cancer prevention services. At the same time, we still need to explore new treatment methods to improve the survival rate and quality of life of esophageal cancer patients.

### Electronic supplementary material

Below is the link to the electronic supplementary material.

Supplementary Material 1

## Data Availability

All data generated or analyzed during this study are included in this published article [and its supplementary information files].
