# Supplementary material for: Age-period-cohort analysis of incidence, mortality and disability-adjusted life years of esophageal cancer in global, regional and national regions from 1990 to 2019
Source: BMC Public Health. 2024 Jan 17;24:212. doi: 10.1186/s12889-024-17706-8 (PMC10795420; doi:10.1186/s12889-024-17706-8)
Supplement: Supplementary file 1 — Supplementary Material 1 [file 12889_2024_17706_MOESM1_ESM.docx]

Supplementary appendix

A B


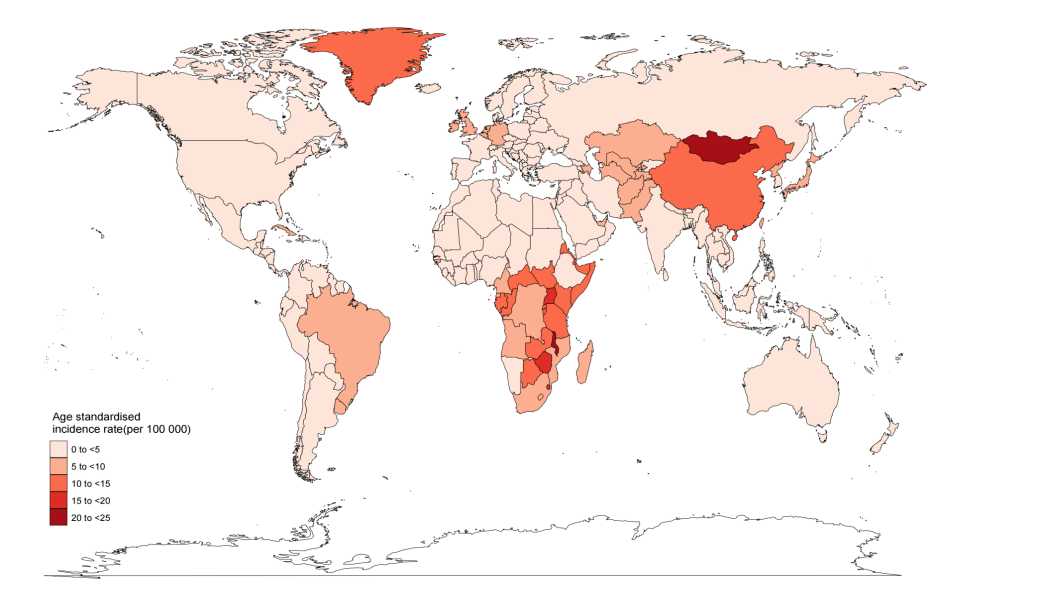

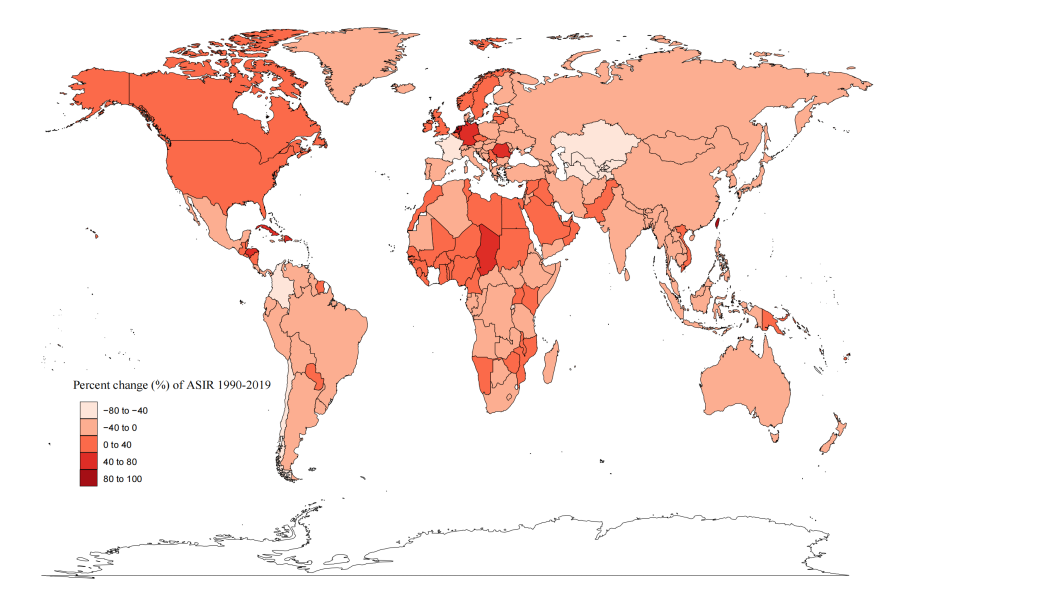


Figure S1 (A)Geographical distribution of age-standardized incidence rates of esophageal cancer in 2019. (B)The percentage change in age-standardized incidence rates of esophageal cancer for 204 countries and territories from 1990 to 2019.

A B


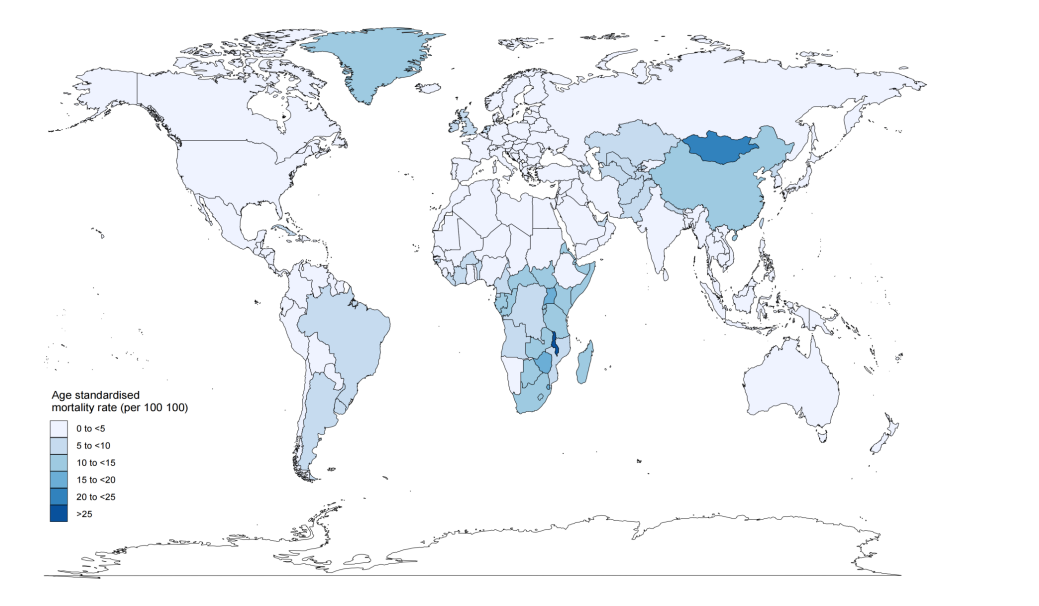

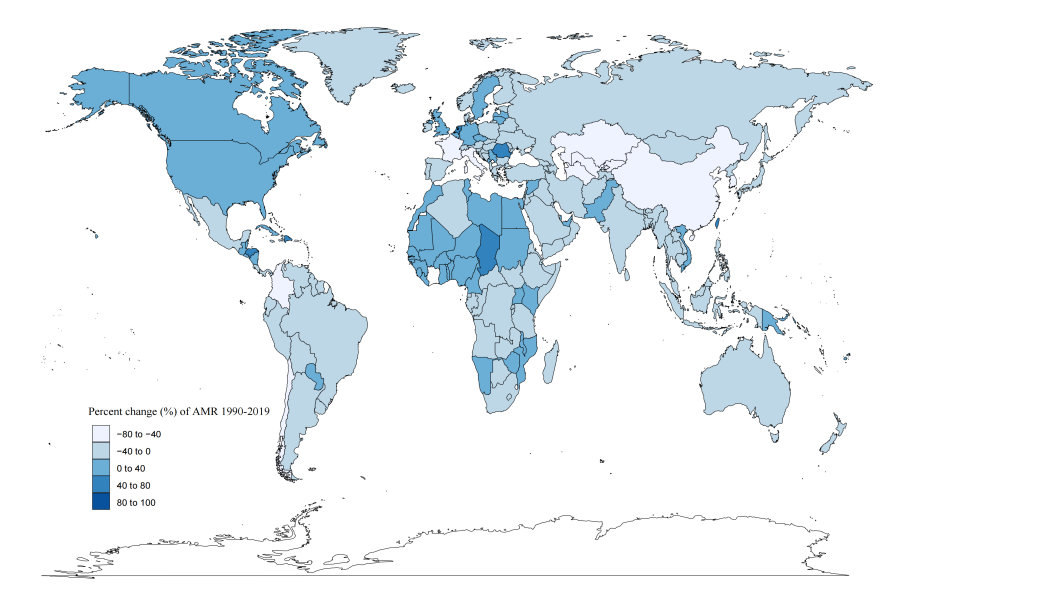


Figure S2 (A)Geographical distribution of age-standardized mortality rates of esophageal cancer in 2019. (B)The percentage change in age-standardized mortality rates of esophageal cancer for 204 countries and territories from 1990 to 2019.


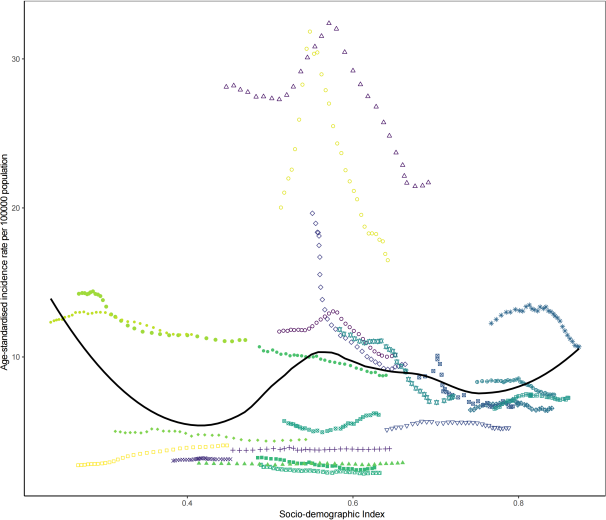

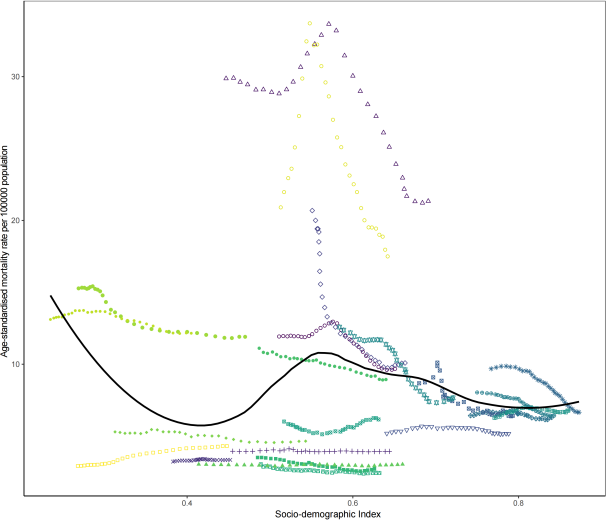

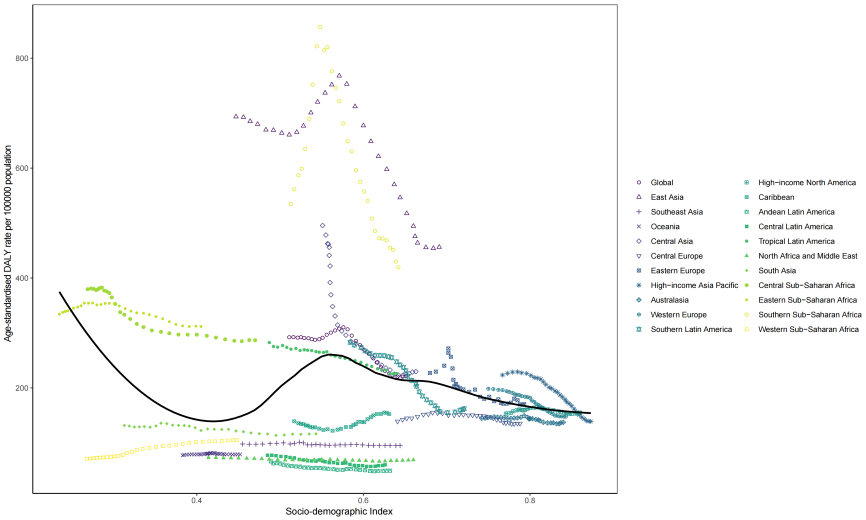


A


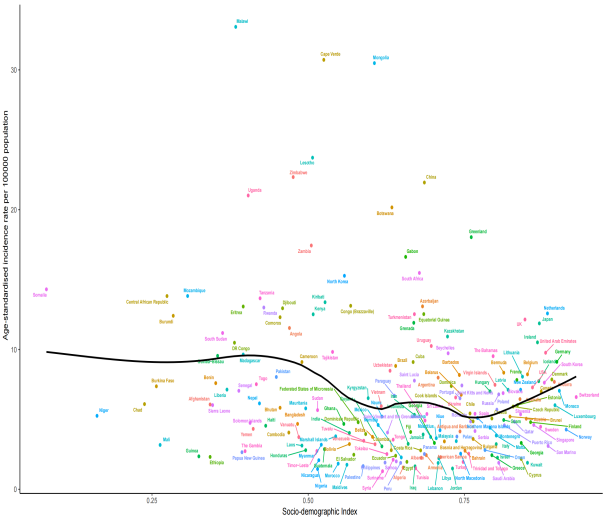

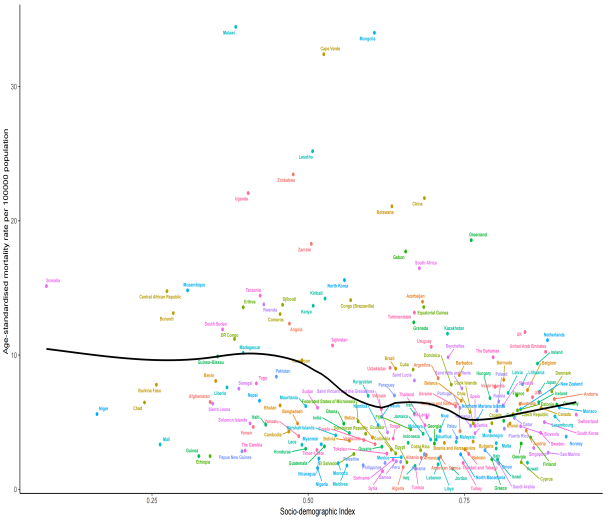

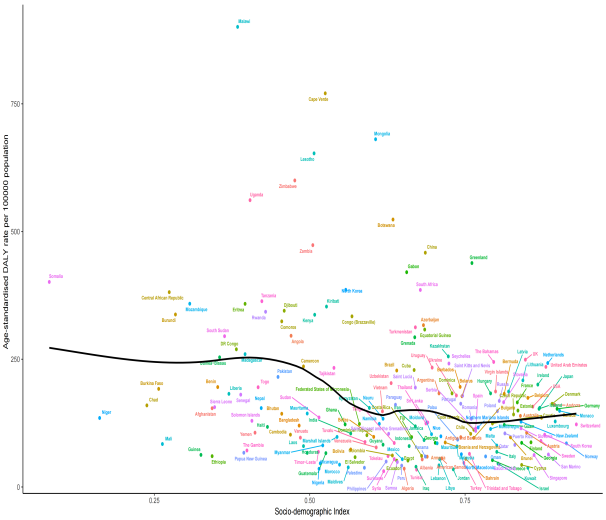


B

Figure S3: Age-standardized incidence, mortality and DALY rates for male esophageal cancer for 21 GBD regions (A) and 195 countries and territories (B) by Socio-demographic index, 1990–2019.


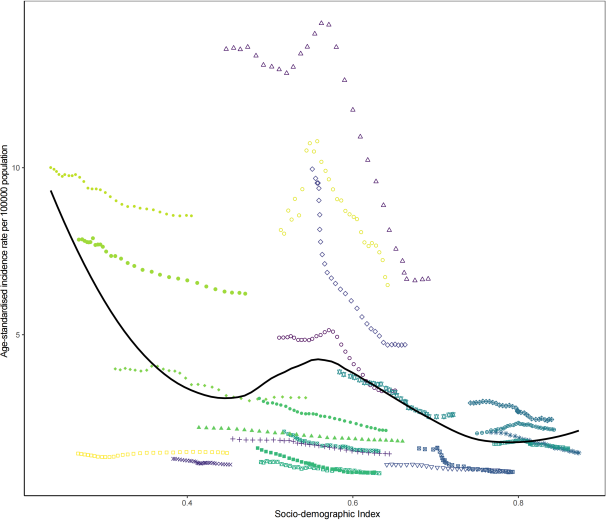

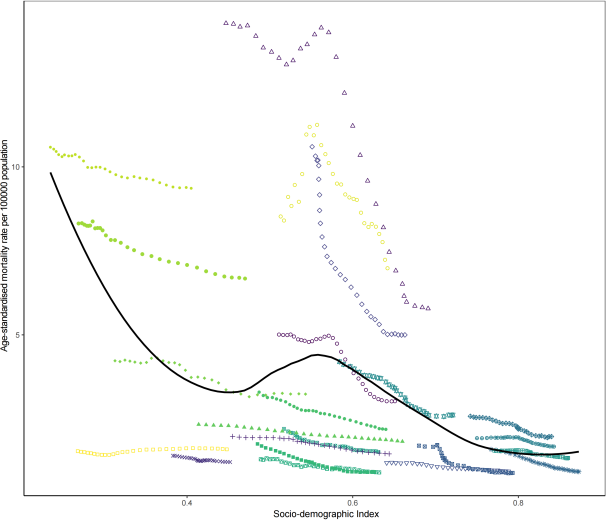

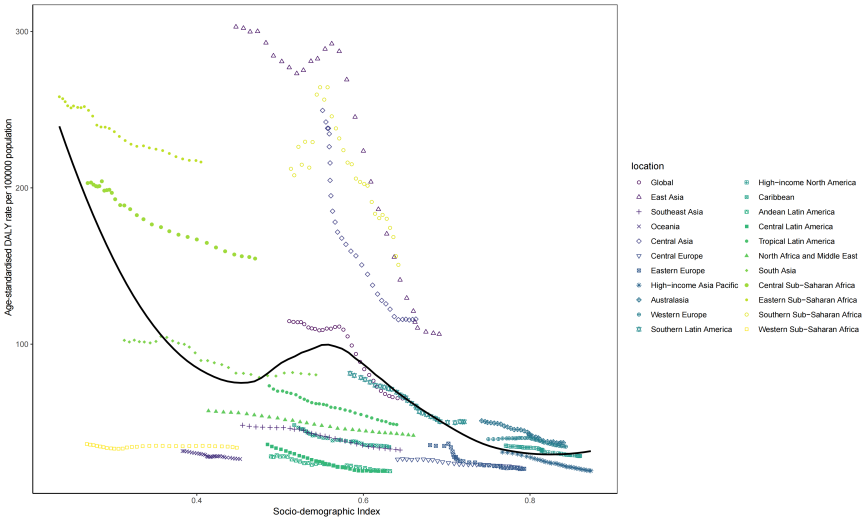


A


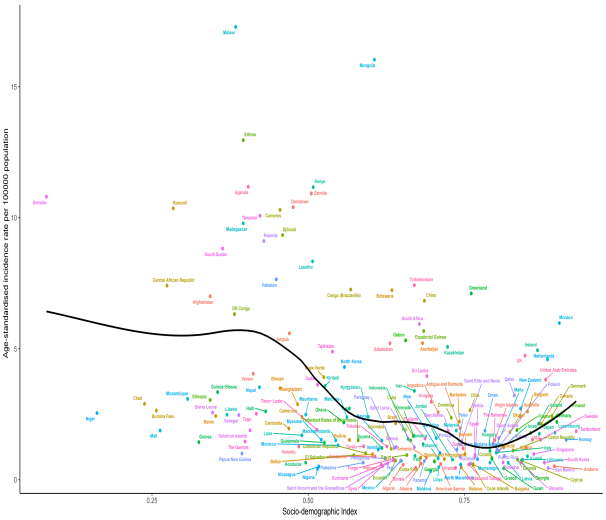

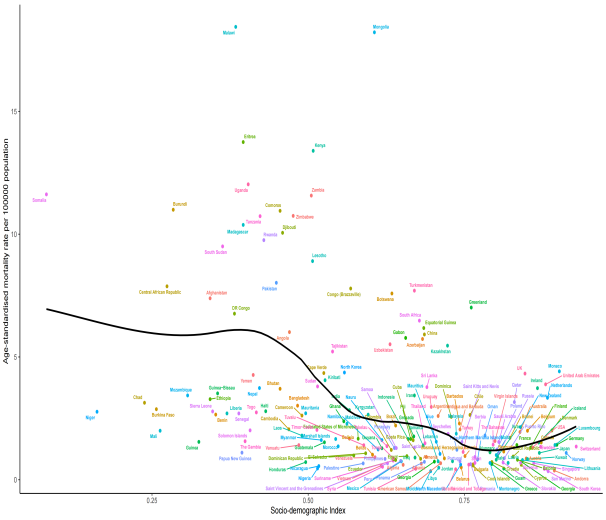

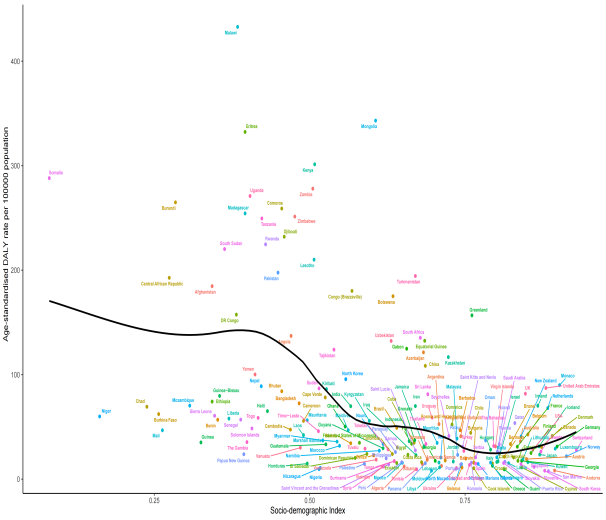


B

Figure S4: Age-standardized incidence, mortality and DALY rates for female esophageal cancer for 21 GBD regions (A) and 195 countries and territories (B) by Socio-demographic Index, 1990–2019.


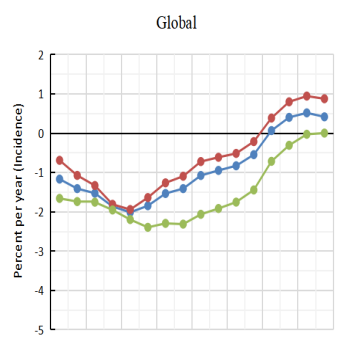

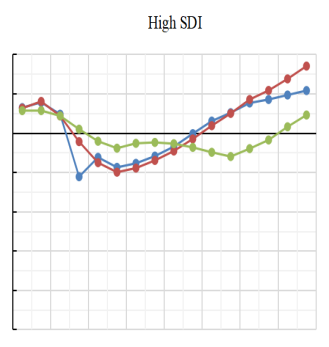

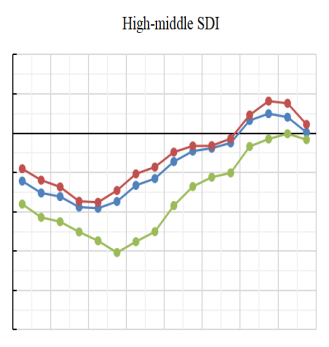

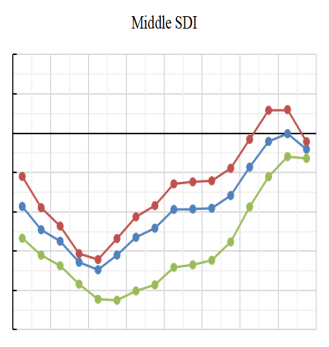

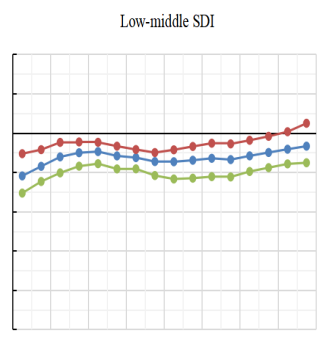

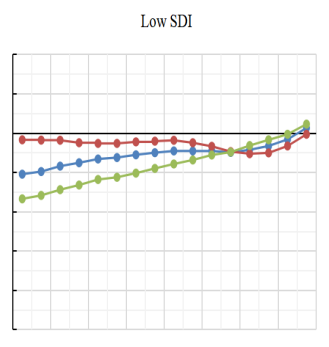


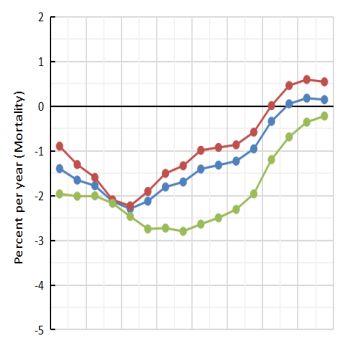

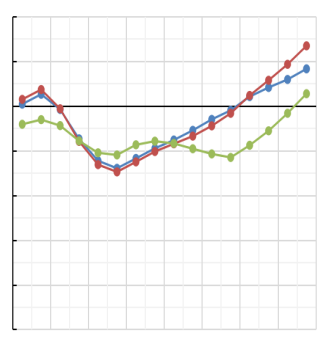

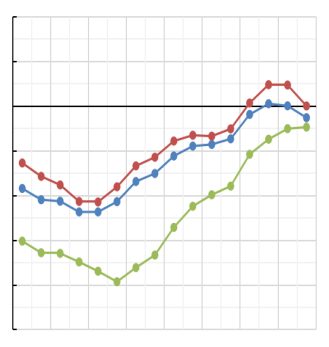

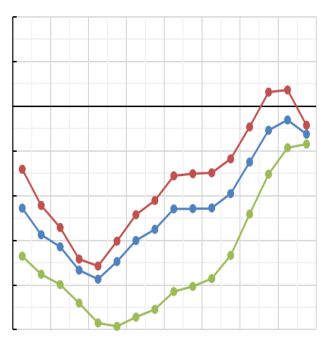

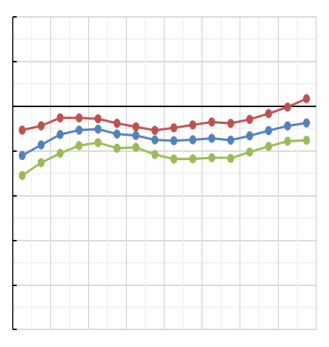

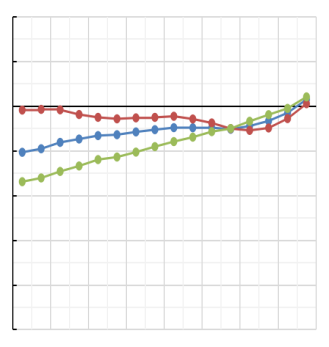


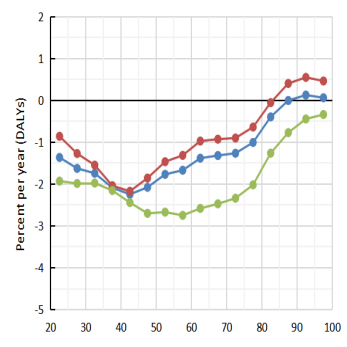

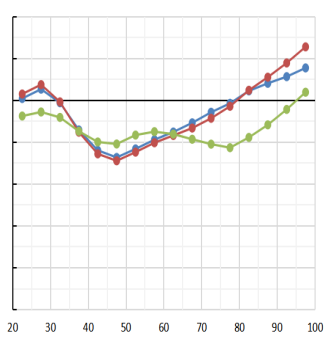

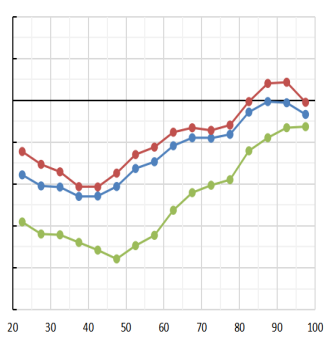

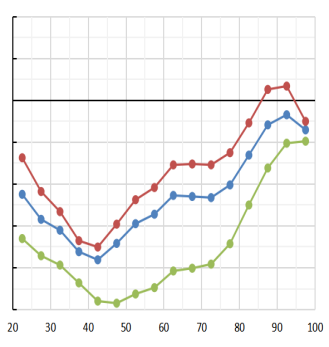

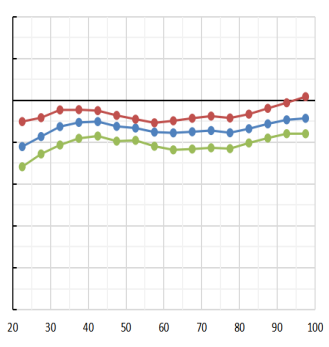

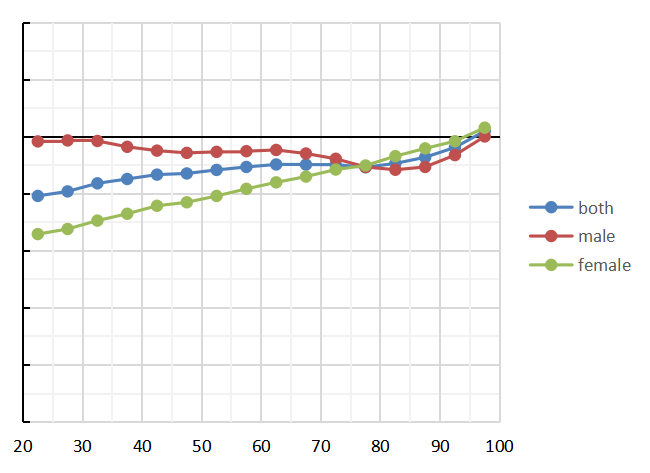


Age，year

Figure S5: Results of local drift of incidence, mortality and DALYs of esophageal cancer globally and across countries and regions with different SDI in the total and male and female populations, 1990–2019.

Table S 1. Age, period, and cohort coefficients and 95% confidence intervals for esophageal cancer incidence, mortality and DALY rates globally and in different SDI regions from 1990 to 2019.

| Index | Variable | | Global | | | High-SDI | | | High-middle SDI | | | Middle SDI | | | Low-middle SDI | | | Low SDI | | |
| --- | --- | --- | --- | --- | --- | --- | --- | --- | --- | --- | --- | --- | --- | --- | --- | --- | --- | --- | --- | --- |
|  |  |  | male | female | both | male | female | both | male | female | both | male | female | both | male | female | both | male | female | both |
| Incidence | Age | 20-24 | 0.18(0.14-0.21) | 0.26(0.21-0.32) | 0.26(0.21-0.32) | 0.06(0.06-0.08) | 0.03(0.02-0.04) | 0.05(0.04-0.06) | 0.19(0.14-0.25) | 0.24(0.16-0.35) | 0.18(0.14-0.24) | 0.3(0.22-0.41) | 0.56(0.41-0.76) | 0.37(0.29-0.48) | 0.12(0.11-0.13) | 0.23(0.21-0.25) | 0.17(0.16-0.18) | 0.09(0.08-0.1) | 0.2(0.18-0.21) | 0.14(0.13-0.14) |
|  |  | 25-29 | 0.29(0.25-0.34) | 0.4(0.34-0.47) | 0.4(0.34-0.47) | 0.14(0.12-0.15) | 0.06(0.05-0.06) | 0.1(0.09-0.11) | 0.28(0.22-0.34) | 0.33(0.24-0.45) | 0.27(0.22-0.33) | 0.46(0.36-0.58) | 0.76(0.6-0.97) | 0.54(0.45-0.65) | 0.22(0.2-0.23) | 0.37(0.34-0.4) | 0.28(0.27-0.3) | 0.17(0.16-0.18) | 0.41(0.39-0.43) | 0.27(0.26-0.29) |
|  |  | 30-34 | 0.75(0.69-0.83) | 0.6(0.53-0.68) | 0.6(0.53-0.68) | 0.37(0.35-0.4) | 0.1(0.09-0.11) | 0.24(0.22-0.26) | 0.79(0.69-0.9) | 0.51(0.41-0.64) | 0.61(0.54-0.7) | 1.24(1.08-1.43) | 1.15(0.95-1.38) | 1.13(0.99-1.28) | 0.47(0.45-0.5) | 0.52(0.48-0.55) | 0.49(0.47-0.51) | 0.42(0.4-0.44) | 0.66(0.63-0.69) | 0.52(0.51-0.54) |
|  |  | 35-39 | 2(1.89-2.12) | 1.03(0.94-1.13) | 1.03(0.94-1.13) | 1.09(1.05-1.13) | 0.24(0.22-0.26) | 0.67(0.64-0.7) | 2.1(1.94-2.28) | 0.89(0.75-1.06) | 1.47(1.35-1.61) | 3.24(2.97-3.53) | 1.74(1.51-2.01) | 2.46(2.26-2.69) | 1.19(1.15-1.23) | 0.92(0.88-0.97) | 1.06(1.03-1.09) | 1.16(1.13-1.2) | 1.3(1.26-1.35) | 1.22(1.19-1.24) |
|  |  | 40-44 | 5.47(5.26-5.68) | 2.12(1.98-2.26) | 2.12(1.98-2.26) | 3.28(3.2-3.35) | 0.61(0.58-0.64) | 1.96(1.91-2.01) | 5.99(5.7-6.3) | 1.74(1.54-1.96) | 3.89(3.67-4.11) | 8.75(8.27-9.26) | 3.57(3.22-3.95) | 6.22(5.88-6.59) | 2.85(2.78-2.92) | 1.89(1.82-1.96) | 2.39(2.34-2.44) | 3.26(3.19-3.33) | 2.66(2.59-2.73) | 2.96(2.91-3.01) |
|  |  | 45-49 | 11.77(11.45-12.11) | 4.24(4.04-4.46) | 4.24(4.04-4.46) | 8.52(8.4-8.65) | 1.5(1.45-1.54) | 5.03(4.94-5.12) | 13.74(13.26-14.24) | 3.61(3.31-3.93) | 8.73(8.38-9.08) | 16.91(16.21-17.65) | 6.78(6.29-7.31) | 11.96(11.45-12.49) | 6.38(6.25-6.5) | 3.86(3.75-3.98) | 5.17(5.09-5.26) | 7.09(6.97-7.21) | 5.41(5.3-5.52) | 6.27(6.19-6.36) |
|  |  | 50-54 | 21.7(21.22-22.19) | 7.23(6.95-7.53) | 7.23(6.95-7.53) | 16.46(16.28-16.65) | 2.93(2.87-3.01) | 9.69(9.56-9.83) | 25.7(24.98-26.45) | 6.28(5.88-6.71) | 16(15.5-16.52) | 29.47(28.46-30.51) | 10.76(10.13-11.43) | 20.32(19.62-21.04) | 12.2(12.01-12.39) | 6.78(6.62-6.95) | 9.58(9.45-9.71) | 14.4(14.2-14.59) | 10.14(9.97-10.31) | 12.33(12.2-12.46) |
|  |  | 55-59 | 33.89(33.24-34.56) | 10.53(10.17-10.9) | 10.53(10.17-10.9) | 27.38(27.12-27.64) | 4.79(4.7-4.89) | 15.97(15.79-16.16) | 40.68(39.68-41.69) | 9.44(8.93-9.97) | 24.8(24.13-25.48) | 44.7(43.37-46.07) | 15.42(14.65-16.23) | 30.23(29.33-31.16) | 19.08(18.82-19.35) | 9.44(9.23-9.65) | 14.32(14.15-14.5) | 21.04(20.78-21.3) | 14.88(14.65-15.1) | 18.02(17.84-18.19) |
|  |  | 60-64 | 45.53(44.71-46.36) | 14.45(14.01-14.91) | 14.45(14.01-14.91) | 38.54(38.21-38.88) | 6.79(6.67-6.91) | 22.32(22.09-22.56) | 54.52(53.26-55.8) | 13.61(12.95-14.29) | 33.24(32.41-34.09) | 59.78(58.12-61.48) | 22.07(21.08-23.1) | 40.84(39.72-41.99) | 25.12(24.79-25.46) | 11.28(11.04-11.53) | 18.15(17.94-18.37) | 27.46(27.14-27.79) | 18.43(18.17-18.7) | 23(22.79-23.21) |
|  |  | 65-69 | 57.45(56.41-58.5) | 19.08(18.51-19.67) | 19.08(18.51-19.67) | 49.98(49.56-50.4) | 9.15(9-9.31) | 28.74(28.45-29.04) | 65.87(64.34-67.43) | 18.67(17.8-19.58) | 40.66(39.64-41.71) | 77.91(75.76-80.13) | 29.56(28.25-30.92) | 53.22(51.77-54.71) | 30.41(30-30.82) | 14.16(13.87-14.46) | 22.09(21.83-22.35) | 32.82(32.42-33.22) | 22.01(21.68-22.34) | 27.43(27.17-27.68) |
|  |  | 70-74 | 70.54(69.21-71.89) | 24.22(23.49-24.98) | 24.22(23.49-24.98) | 60.73(60.21-61.26) | 12.25(12.04-12.46) | 34.82(34.45-35.18) | 81.15(79.18-83.16) | 24.53(23.41-25.72) | 50.04(48.74-51.36) | 97.88(95.03-100.8) | 36.99(35.34-38.72) | 66.17(64.3-68.1) | 33.71(33.22-34.21) | 18.26(17.87-18.66) | 25.66(25.34-25.98) | 37.02(36.53-37.52) | 27.43(27.01-27.86) | 32.16(31.84-32.49) |
|  |  | 75-79 | 76.44(74.66-78.25) | 26.35(25.42-27.32) | 26.35(25.42-27.32) | 67.2(66.5-67.92) | 15.74(15.44-16.05) | 38.68(38.2-39.17) | 88.56(85.93-91.27) | 27.68(26.2-29.24) | 53.77(52.1-55.49) | 105.11(101.37-108.99) | 39.03(37.01-41.16) | 69.73(67.36-72.19) | 36.29(35.62-36.97) | 20.8(20.25-21.36) | 28.04(27.6-28.48) | 35.72(35.09-36.35) | 27.56(27.01-28.11) | 31.49(31.08-31.91) |
|  |  | 80-84 | 82.56(80.3-84.89) | 28.78(27.66-29.94) | 28.78(27.66-29.94) | 77.9(76.98-78.83) | 21.44(21.01-21.87) | 45.21(44.59-45.84) | 97.72(94.31-101.24) | 31.64(29.81-33.57) | 58.19(56.13-60.32) | 109.27(104.58-114.18) | 40.87(38.52-43.37) | 71.57(68.72-74.53) | 36.48(35.63-37.35) | 20.54(19.89-21.21) | 27.76(27.22-28.31) | 35.81(35-36.65) | 32.04(31.29-32.82) | 33.85(33.3-34.41) |
|  |  | 85-89 | 83.16(80.05-86.4) | 28.09(26.77-29.48) | 28.09(26.77-29.48) | 78.6(77.43-79.8) | 25.39(24.84-25.95) | 46.48(45.72-47.25) | 102.79(97.91-107.92) | 31.39(29.21-33.73) | 57.06(54.44-59.81) | 109.44(102.76-116.55) | 37.09(34.36-40.03) | 67(63.38-70.84) | 37.81(36.54-39.13) | 22.58(21.64-23.55) | 29.3(28.51-30.11) | 35.61(34.4-36.86) | 37.47(36.3-38.67) | 36.78(35.94-37.64) |
|  |  | 90-94 | 59.34(55.07-63.95) | 23.99(22.34-25.77) | 23.99(22.34-25.77) | 73.89(72.18-75.64) | 25.03(24.38-25.7) | 43.05(42.07-44.06) | 65.67(59.07-73.01) | 28.47(25.58-31.67) | 42.06(38.64-45.78) | 60.11(51.7-69.88) | 29.84(26.33-33.82) | 44.92(40.27-50.12) | 29.45(27.52-31.53) | 18.11(16.82-19.51) | 22.97(21.83-24.17) | 28.4(26.48-30.46) | 29.33(27.71-31.04) | 29.09(27.84-30.39) |
|  |  | ≥95 | 42.43(35.57-50.62) | 18.43(16.07-21.15) | 18.43(16.07-21.15) | 60.02(57.23-62.95) | 22.08(21.22-22.97) | 35.71(34.26-37.21) | 41.46(31.05-55.36) | 21.99(17.54-27.58) | 30.15(24.64-36.89) | 30(19.61-45.88) | 19.83(14.88-26.42) | 27.39(20.84-36.01) | 24.24(20.69-28.39) | 14.42(12.35-16.83) | 18.37(16.41-20.55) | 23.66(19.75-28.35) | 25.04(22.16-28.29) | 24.7(22.33-27.32) |
|  | Period | 1990-1994 | 0.97(0.95-1) | 1.02(0.99-1.05) | 1.02(0.99-1.05) | 0.96(0.95-0.97) | 0.95(0.94-0.97) | 0.95(0.94-0.96) | 0.96(0.93-0.99) | 1.05(0.99-1.1) | 0.97(0.95-1) | 1.01(0.97-1.04) | 1.08(1.04-1.14) | 1.03(1-1.06) | 0.99(0.97-1.01) | 1.05(1.03-1.07) | 1.02(1-1.03) | 0.98(0.97-1) | 1.06(1.04-1.08) | 1.02(1.01-1.03) |
|  |  | 1995-1999 | 0.95(0.93-0.97) | 0.97(0.95-1) | 0.97(0.95-1) | 0.98(0.97-0.99) | 0.98(0.97-1) | 0.98(0.97-0.99) | 0.93(0.91-0.96) | 0.97(0.93-1.02) | 0.94(0.92-0.97) | 0.95(0.92-0.98) | 0.99(0.95-1.03) | 0.96(0.93-0.99) | 0.99(0.98-1.01) | 1.03(1.01-1.05) | 1.01(1-1.02) | 1(0.99-1.01) | 1.04(1.03-1.06) | 1.02(1.01-1.03) |
|  |  | 2000-2004 | 1(1-1) | 1(1-1) | 1(1-1) | 1(1-1) | 1(1-1) | 1(1-1) | 1(1-1) | 1(1-1) | 1(1-1) | 1(1-1) | 1(1-1) | 1(1-1) | 1(1-1) | 1(1-1) | 1(1-1) | 1(1-1) | 1(1-1) | 1(1-1) |
|  |  | 2005-2009 | 0.96(0.94-0.98) | 0.89(0.87-0.91) | 0.89(0.87-0.91) | 1(0.99-1.01) | 0.98(0.96-0.99) | 1(0.99-1.01) | 0.95(0.93-0.97) | 0.88(0.85-0.92) | 0.94(0.91-0.96) | 0.93(0.9-0.96) | 0.83(0.8-0.86) | 0.89(0.87-0.92) | 0.98(0.97-1) | 0.92(0.91-0.94) | 0.96(0.95-0.97) | 0.97(0.96-0.98) | 0.94(0.92-0.95) | 0.96(0.95-0.96) |
|  |  | 2010-2014 | 0.85(0.84-0.87) | 0.73(0.71-0.75) | 0.73(0.71-0.75) | 0.97(0.96-0.98) | 0.94(0.93-0.96) | 0.97(0.96-0.99) | 0.85(0.83-0.87) | 0.73(0.69-0.76) | 0.82(0.8-0.84) | 0.77(0.74-0.79) | 0.59(0.56-0.62) | 0.7(0.68-0.72) | 0.94(0.92-0.95) | 0.85(0.83-0.86) | 0.9(0.89-0.91) | 0.95(0.93-0.96) | 0.89(0.88-0.91) | 0.92(0.91-0.93) |
|  |  | 2015-2019 | 0.8(0.78-0.82) | 0.67(0.65-0.7) | 0.67(0.65-0.7) | 0.96(0.94-0.97) | 0.92(0.9-0.94) | 0.96(0.94-0.97) | 0.81(0.79-0.84) | 0.68(0.65-0.72) | 0.79(0.76-0.81) | 0.67(0.65-0.7) | 0.49(0.47-0.52) | 0.6(0.58-0.63) | 0.93(0.91-0.94) | 0.84(0.82-0.86) | 0.88(0.87-0.9) | 0.93(0.91-0.94) | 0.88(0.87-0.9) | 0.91(0.9-0.92) |
|  | Cohort | 1895 | 0.9(0.45-1.8) | 1.52(0.95-2.45) | 1.52(0.95-2.45) | 0.62(0.51-0.75) | 1(0.88-1.14) | 0.71(0.61-0.82) | 1.03(0.37-2.84) | 1.35(0.63-2.89) | 1.08(0.54-2.16) | 1.48(0.33-6.68) | 2.64(1-6.99) | 1.8(0.7-4.62) | 1.02(0.55-1.87) | 1.61(0.94-2.74) | 1.29(0.86-1.94) | 1.11(0.6-2.06) | 1.07(0.7-1.63) | 1.08(0.76-1.53) |
|  |  | 1900 | 0.92(0.73-1.17) | 1.48(1.22-1.79) | 1.48(1.22-1.79) | 0.7(0.65-0.75) | 1.04(0.98-1.1) | 0.75(0.7-0.8) | 0.92(0.66-1.29) | 1.25(0.93-1.66) | 0.99(0.77-1.28) | 1.22(0.75-1.97) | 2.38(1.66-3.41) | 1.58(1.13-2.21) | 1.06(0.86-1.3) | 1.53(1.24-1.88) | 1.27(1.09-1.47) | 1.15(0.93-1.41) | 1.13(0.97-1.33) | 1.14(1-1.29) |
|  |  | 1905 | 0.93(0.84-1.02) | 1.49(1.35-1.64) | 1.49(1.35-1.64) | 0.79(0.76-0.82) | 1.09(1.06-1.13) | 0.82(0.79-0.84) | 0.87(0.76-0.99) | 1.24(1.07-1.44) | 0.95(0.85-1.06) | 1.07(0.89-1.28) | 2.32(1.96-2.74) | 1.46(1.27-1.69) | 1.05(0.96-1.15) | 1.47(1.33-1.62) | 1.24(1.16-1.32) | 1.15(1.06-1.26) | 1.15(1.07-1.24) | 1.15(1.08-1.22) |
|  |  | 1910 | 0.96(0.91-1.02) | 1.44(1.35-1.54) | 1.44(1.35-1.54) | 0.83(0.81-0.85) | 1.1(1.07-1.13) | 0.84(0.82-0.86) | 0.88(0.82-0.95) | 1.17(1.06-1.29) | 0.92(0.86-0.99) | 1.17(1.06-1.29) | 2.27(2.05-2.52) | 1.5(1.38-1.63) | 1.07(1.02-1.12) | 1.4(1.32-1.49) | 1.21(1.17-1.26) | 1.15(1.1-1.21) | 1.15(1.1-1.2) | 1.15(1.11-1.19) |
|  |  | 1915 | 1.06(1.02-1.1) | 1.52(1.44-1.6) | 1.52(1.44-1.6) | 0.9(0.88-0.91) | 1.13(1.11-1.16) | 0.89(0.88-0.91) | 0.98(0.93-1.04) | 1.24(1.15-1.34) | 1.01(0.96-1.06) | 1.27(1.2-1.36) | 2.28(2.12-2.47) | 1.57(1.48-1.66) | 1.1(1.07-1.14) | 1.38(1.33-1.44) | 1.22(1.19-1.26) | 1.14(1.11-1.18) | 1.16(1.13-1.2) | 1.15(1.13-1.18) |
|  |  | 1920 | 1.12(1.08-1.15) | 1.51(1.45-1.58) | 1.51(1.45-1.58) | 0.95(0.93-0.96) | 1.11(1.09-1.14) | 0.92(0.91-0.94) | 1.07(1.03-1.11) | 1.29(1.21-1.37) | 1.08(1.04-1.13) | 1.31(1.25-1.38) | 2.17(2.04-2.31) | 1.56(1.49-1.63) | 1.08(1.05-1.11) | 1.32(1.28-1.37) | 1.18(1.15-1.2) | 1.1(1.07-1.12) | 1.15(1.12-1.17) | 1.12(1.1-1.14) |
|  |  | 1925 | 1.12(1.1-1.15) | 1.44(1.38-1.49) | 1.44(1.38-1.49) | 0.99(0.98-1) | 1.08(1.06-1.1) | 0.96(0.95-0.98) | 1.04(1.01-1.07) | 1.2(1.13-1.27) | 1.04(1.01-1.08) | 1.3(1.25-1.35) | 2(1.9-2.12) | 1.5(1.45-1.56) | 1.05(1.03-1.07) | 1.24(1.2-1.28) | 1.13(1.11-1.15) | 1.06(1.04-1.08) | 1.12(1.1-1.15) | 1.09(1.07-1.1) |
|  |  | 1930 | 1.1(1.08-1.13) | 1.34(1.3-1.39) | 1.34(1.3-1.39) | 1.03(1.02-1.04) | 1.04(1.02-1.06) | 1.01(0.99-1.02) | 1.03(1-1.05) | 1.15(1.09-1.22) | 1.04(1.01-1.07) | 1.22(1.18-1.26) | 1.71(1.63-1.8) | 1.36(1.32-1.41) | 1.04(1.02-1.06) | 1.17(1.14-1.2) | 1.09(1.08-1.11) | 1.03(1.01-1.05) | 1.1(1.08-1.12) | 1.06(1.05-1.08) |
|  |  | 1935 | 1.07(1.05-1.1) | 1.22(1.18-1.26) | 1.22(1.18-1.26) | 1.03(1.02-1.04) | 1.01(0.99-1.03) | 1.01(1-1.02) | 1.01(0.99-1.04) | 1.1(1.05-1.16) | 1.03(1-1.05) | 1.14(1.1-1.17) | 1.42(1.35-1.49) | 1.22(1.18-1.25) | 1.04(1.03-1.06) | 1.11(1.09-1.14) | 1.07(1.05-1.08) | 1.03(1.02-1.05) | 1.07(1.05-1.08) | 1.05(1.03-1.06) |
|  |  | 1940 | 1.01(0.99-1.03) | 1.05(1.02-1.09) | 1.05(1.02-1.09) | 1.01(1-1.02) | 0.98(0.96-1) | 1(0.99-1.02) | 0.97(0.94-1) | 0.96(0.91-1.02) | 0.96(0.93-0.98) | 1.03(1-1.06) | 1.14(1.09-1.2) | 1.06(1.03-1.09) | 1.03(1.01-1.04) | 1.05(1.02-1.07) | 1.03(1.02-1.04) | 1.01(1-1.03) | 1.03(1.01-1.05) | 1.02(1.01-1.03) |
|  |  | 1945 | 1(1-1) | 1(1-1) | 1(1-1) | 1(1-1) | 1(1-1) | 1(1-1) | 1(1-1) | 1(1-1) | 1(1-1) | 1(1-1) | 1(1-1) | 1(1-1) | 1(1-1) | 1(1-1) | 1(1-1) | 1(1-1) | 1(1-1) | 1(1-1) |
|  |  | 1950 | 0.97(0.95-0.99) | 0.9(0.86-0.93) | 0.9(0.86-0.93) | 0.96(0.95-0.97) | 0.98(0.96-1) | 0.96(0.95-0.98) | 0.95(0.93-0.98) | 0.83(0.78-0.88) | 0.92(0.89-0.95) | 0.97(0.94-1) | 0.88(0.83-0.93) | 0.94(0.91-0.97) | 0.96(0.94-0.97) | 0.92(0.9-0.94) | 0.94(0.93-0.96) | 0.99(0.98-1) | 0.94(0.93-0.96) | 0.97(0.96-0.98) |
|  |  | 1955 | 0.91(0.89-0.93) | 0.79(0.75-0.82) | 0.79(0.75-0.82) | 0.91(0.9-0.92) | 0.96(0.94-0.99) | 0.92(0.91-0.93) | 0.89(0.87-0.92) | 0.71(0.66-0.76) | 0.85(0.82-0.88) | 0.86(0.83-0.89) | 0.69(0.65-0.74) | 0.81(0.78-0.84) | 0.95(0.93-0.96) | 0.88(0.86-0.9) | 0.92(0.91-0.93) | 0.99(0.98-1.01) | 0.9(0.89-0.92) | 0.95(0.94-0.96) |
|  |  | 1960 | 0.78(0.76-0.8) | 0.65(0.62-0.69) | 0.65(0.62-0.69) | 0.86(0.85-0.87) | 0.94(0.92-0.97) | 0.88(0.86-0.89) | 0.79(0.77-0.82) | 0.57(0.52-0.62) | 0.74(0.72-0.77) | 0.67(0.64-0.7) | 0.49(0.46-0.54) | 0.62(0.59-0.64) | 0.93(0.91-0.95) | 0.86(0.84-0.89) | 0.9(0.88-0.91) | 0.97(0.96-0.99) | 0.85(0.83-0.87) | 0.92(0.91-0.93) |
|  |  | 1965 | 0.76(0.73-0.79) | 0.62(0.58-0.66) | 0.62(0.58-0.66) | 0.82(0.8-0.83) | 0.93(0.9-0.97) | 0.84(0.82-0.86) | 0.78(0.75-0.82) | 0.53(0.47-0.59) | 0.73(0.69-0.76) | 0.63(0.6-0.67) | 0.44(0.39-0.48) | 0.57(0.54-0.6) | 0.93(0.91-0.95) | 0.84(0.81-0.87) | 0.89(0.87-0.91) | 0.95(0.93-0.97) | 0.8(0.78-0.82) | 0.89(0.87-0.9) |
|  |  | 1970 | 0.67(0.64-0.7) | 0.55(0.51-0.6) | 0.55(0.51-0.6) | 0.79(0.76-0.81) | 0.91(0.86-0.96) | 0.81(0.78-0.84) | 0.69(0.65-0.73) | 0.47(0.4-0.54) | 0.64(0.6-0.69) | 0.52(0.49-0.56) | 0.36(0.31-0.41) | 0.47(0.44-0.51) | 0.91(0.89-0.94) | 0.77(0.74-0.8) | 0.85(0.83-0.87) | 0.94(0.92-0.96) | 0.75(0.73-0.78) | 0.86(0.84-0.87) |
|  |  | 1975 | 0.59(0.55-0.64) | 0.51(0.46-0.57) | 0.51(0.46-0.57) | 0.82(0.78-0.85) | 0.95(0.87-1.03) | 0.85(0.8-0.89) | 0.6(0.54-0.66) | 0.41(0.33-0.51) | 0.56(0.5-0.62) | 0.42(0.37-0.47) | 0.3(0.25-0.36) | 0.38(0.34-0.43) | 0.9(0.87-0.94) | 0.76(0.72-0.81) | 0.84(0.81-0.87) | 0.94(0.91-0.97) | 0.7(0.68-0.73) | 0.83(0.81-0.85) |
|  |  | 1980 | 0.58(0.52-0.65) | 0.47(0.4-0.54) | 0.47(0.4-0.54) | 0.88(0.82-0.95) | 1(0.88-1.13) | 0.91(0.84-0.99) | 0.59(0.5-0.69) | 0.36(0.27-0.49) | 0.53(0.45-0.63) | 0.4(0.33-0.48) | 0.25(0.19-0.32) | 0.35(0.29-0.41) | 0.89(0.84-0.95) | 0.72(0.67-0.77) | 0.81(0.77-0.85) | 0.93(0.89-0.98) | 0.64(0.61-0.68) | 0.79(0.76-0.81) |
|  |  | 1985 | 0.59(0.5-0.7) | 0.42(0.35-0.52) | 0.42(0.35-0.52) | 0.95(0.85-1.07) | 1.04(0.87-1.25) | 0.98(0.86-1.12) | 0.6(0.47-0.77) | 0.33(0.22-0.5) | 0.52(0.4-0.67) | 0.4(0.3-0.53) | 0.22(0.15-0.31) | 0.33(0.25-0.42) | 0.88(0.81-0.95) | 0.67(0.61-0.73) | 0.77(0.72-0.82) | 0.92(0.86-0.99) | 0.59(0.55-0.63) | 0.74(0.71-0.78) |
|  |  | 1990 | 0.57(0.43-0.74) | 0.4(0.3-0.53) | 0.4(0.3-0.53) | 0.95(0.78-1.16) | 1.03(0.79-1.36) | 0.97(0.78-1.21) | 0.56(0.37-0.85) | 0.31(0.17-0.57) | 0.49(0.32-0.73) | 0.39(0.25-0.6) | 0.2(0.12-0.33) | 0.3(0.21-0.45) | 0.82(0.72-0.93) | 0.6(0.53-0.68) | 0.71(0.65-0.77) | 0.9(0.81-1.01) | 0.54(0.49-0.59) | 0.69(0.65-0.74) |
|  |  | 1995 | 0.54(0.34-0.85) | 0.36(0.23-0.58) | 0.36(0.23-0.58) | 0.88(0.61-1.26) | 1.04(0.66-1.66) | 0.92(0.62-1.37) | 0.52(0.25-1.08) | 0.3(0.11-0.81) | 0.46(0.23-0.92) | 0.37(0.18-0.78) | 0.18(0.08-0.4) | 0.28(0.15-0.53) | 0.81(0.66-0.99) | 0.53(0.43-0.64) | 0.65(0.56-0.75) | 0.91(0.77-1.07) | 0.5(0.43-0.58) | 0.67(0.6-0.74) |
| Deaths | Age | 20-24 | 0.13(0.11-0.17) | 0.19(0.15-0.23) | 0.15(0.13-0.18) | 0.04(0.04-0.05) | 0.02(0.02-0.02) | 0.03(0.03-0.04) | 0.14(0.1-0.2) | 0.19(0.13-0.29) | 0.14(0.1-0.19) | 0.23(0.16-0.33) | 0.43(0.31-0.59) | 0.28(0.21-0.36) | 0.08(0.08-0.09) | 0.16(0.15-0.18) | 0.12(0.11-0.13) | 0.06(0.06-0.07) | 0.14(0.13-0.15) | 0.1(0.09-0.1) |
|  |  | 25-29 | 0.23(0.2-0.27) | 0.31(0.27-0.36) | 0.26(0.23-0.29) | 0.1(0.09-0.11) | 0.04(0.03-0.04) | 0.07(0.06-0.08) | 0.22(0.18-0.29) | 0.28(0.2-0.38) | 0.21(0.17-0.26) | 0.38(0.3-0.49) | 0.62(0.48-0.79) | 0.43(0.35-0.52) | 0.17(0.16-0.18) | 0.29(0.26-0.31) | 0.22(0.21-0.23) | 0.13(0.12-0.14) | 0.32(0.3-0.34) | 0.21(0.2-0.22) |
|  |  | 30-34 | 0.66(0.6-0.73) | 0.51(0.45-0.57) | 0.57(0.52-0.61) | 0.3(0.28-0.32) | 0.07(0.06-0.08) | 0.19(0.17-0.2) | 0.7(0.61-0.8) | 0.45(0.36-0.57) | 0.53(0.46-0.61) | 1.12(0.96-1.29) | 0.99(0.82-1.18) | 0.97(0.86-1.11) | 0.41(0.39-0.43) | 0.44(0.41-0.47) | 0.42(0.4-0.44) | 0.34(0.33-0.36) | 0.57(0.55-0.6) | 0.44(0.43-0.46) |
|  |  | 35-39 | 1.79(1.69-1.91) | 0.88(0.81-0.96) | 1.33(1.26-1.41) | 0.87(0.84-0.91) | 0.17(0.16-0.18) | 0.52(0.5-0.55) | 1.91(1.76-2.08) | 0.78(0.66-0.92) | 1.31(1.2-1.43) | 2.98(2.73-3.26) | 1.49(1.3-1.72) | 2.2(2.03-2.4) | 1.07(1.03-1.11) | 0.82(0.77-0.86) | 0.95(0.92-0.98) | 1.01(0.98-1.04) | 1.18(1.14-1.22) | 1.08(1.05-1.1) |
|  |  | 40-44 | 5.13(4.94-5.33) | 1.84(1.73-1.96) | 3.52(3.4-3.65) | 2.68(2.62-2.74) | 0.42(0.4-0.44) | 1.56(1.52-1.6) | 5.69(5.4-6) | 1.52(1.35-1.71) | 3.62(3.43-3.83) | 8.41(7.95-8.89) | 3.05(2.76-3.36) | 5.8(5.5-6.12) | 2.74(2.67-2.81) | 1.78(1.71-1.86) | 2.28(2.23-2.34) | 3.12(3.05-3.19) | 2.56(2.49-2.62) | 2.84(2.79-2.89) |
|  |  | 45-49 | 10.4(10.11-10.7) | 3.45(3.29-3.61) | 7(6.81-7.19) | 6.45(6.35-6.55) | 0.97(0.94-1) | 3.72(3.66-3.79) | 12.32(11.87-12.79) | 2.89(2.66-3.14) | 7.66(7.36-7.96) | 15.31(14.67-15.98) | 5.34(4.97-5.74) | 10.46(10.04-10.9) | 5.9(5.78-6.01) | 3.5(3.39-3.61) | 4.75(4.67-4.83) | 6.62(6.51-6.73) | 4.99(4.89-5.1) | 5.83(5.75-5.91) |
|  |  | 50-54 | 19.37(18.94-19.81) | 6.07(5.86-6.3) | 12.82(12.54-13.09) | 12.78(12.63-12.93) | 2.03(1.99-2.07) | 7.4(7.3-7.5) | 23.31(22.62-24.01) | 5.13(4.82-5.46) | 14.23(13.8-14.67) | 26.89(25.98-27.83) | 8.96(8.47-9.48) | 18.13(17.55-18.73) | 11.38(11.2-11.56) | 6.22(6.05-6.38) | 8.88(8.75-9.01) | 13.68(13.49-13.87) | 9.41(9.25-9.57) | 11.61(11.49-11.74) |
|  |  | 55-59 | 30.28(29.7-30.88) | 8.93(8.66-9.21) | 19.62(19.26-19.98) | 21.03(20.82-21.23) | 3.37(3.31-3.43) | 12.11(11.98-12.25) | 36.99(36.06-37.94) | 7.78(7.39-8.19) | 22.13(21.56-22.72) | 41.03(39.83-42.26) | 13.05(12.45-13.68) | 27.21(26.46-27.98) | 17.98(17.73-18.24) | 8.79(8.59-9.01) | 13.45(13.28-13.63) | 20.27(20.01-20.52) | 14(13.79-14.21) | 17.19(17.03-17.36) |
|  |  | 60-64 | 41.62(40.87-42.38) | 12.48(12.14-12.84) | 26.81(26.36-27.27) | 29.9(29.63-30.17) | 4.91(4.83-4.99) | 17.13(16.96-17.3) | 50.74(49.55-51.96) | 11.34(10.84-11.87) | 30.24(29.52-30.99) | 56.4(54.88-57.97) | 18.97(18.2-19.77) | 37.62(36.67-38.59) | 24.57(24.25-24.9) | 10.97(10.72-11.22) | 17.72(17.51-17.94) | 27.4(27.08-27.73) | 18.07(17.81-18.34) | 22.79(22.58-23) |
|  |  | 65-69 | 53.74(52.78-54.71) | 16.73(16.29-17.19) | 34.57(34-35.15) | 38.87(38.54-39.21) | 6.7(6.6-6.8) | 22.13(21.93-22.35) | 62.82(61.34-64.34) | 15.54(14.87-16.23) | 37.59(36.7-38.51) | 75.8(73.78-77.87) | 25.83(24.81-26.9) | 50.29(49.04-51.57) | 30.98(30.57-31.39) | 14.38(14.07-14.7) | 22.48(22.22-22.76) | 34.11(33.7-34.52) | 22.58(22.25-22.91) | 28.36(28.09-28.62) |
|  |  | 70-74 | 68.06(66.8-69.34) | 21.79(21.22-22.39) | 43.54(42.8-44.29) | 47.58(47.17-48) | 9.13(9-9.26) | 27.04(26.78-27.3) | 80.07(78.12-82.07) | 20.61(19.74-21.52) | 47.44(46.28-48.62) | 99(96.26-101.81) | 33.31(31.98-34.69) | 64.77(63.12-66.47) | 36.15(35.64-36.66) | 19.61(19.17-20.05) | 27.52(27.18-27.87) | 40.62(40.1-41.15) | 29.84(29.39-30.29) | 35.15(34.81-35.5) |
|  |  | 75-79 | 78.48(76.74-80.26) | 25.25(24.48-26.04) | 49.32(48.33-50.33) | 55.96(55.37-56.54) | 12.7(12.5-12.91) | 32.01(31.65-32.37) | 93.27(90.55-96.08) | 24.85(23.67-26.1) | 54.32(52.77-55.92) | 114.05(110.26-117.97) | 37.58(35.88-39.35) | 73.07(70.88-75.34) | 41.88(41.15-42.62) | 24.17(23.53-24.83) | 32.45(31.95-32.96) | 42.21(41.52-42.92) | 32.53(31.92-33.15) | 37.2(36.74-37.66) |
|  |  | 80-84 | 85.84(83.62-88.11) | 27.43(26.53-28.37) | 52.43(51.23-53.66) | 64.33(63.58-65.08) | 16.82(16.54-17.1) | 36.81(36.37-37.26) | 104.84(101.31-108.49) | 28.05(26.62-29.56) | 59.06(57.17-61.01) | 122.56(117.75-127.56) | 40(38.03-42.08) | 76.88(74.23-79.62) | 44.52(43.56-45.49) | 25.3(24.52-26.11) | 34(33.37-34.64) | 44.91(43.98-45.87) | 40.3(39.42-41.2) | 42.5(41.86-43.15) |
|  |  | 85-89 | 95.33(92.14-98.63) | 29.52(28.39-30.7) | 55.43(53.86-57.05) | 75.07(74.04-76.12) | 23.02(22.61-23.42) | 43.55(42.95-44.16) | 122.32(117.01-127.87) | 31.22(29.4-33.15) | 64.4(61.85-67.05) | 134.51(127.42-141.99) | 40.49(38.09-43.05) | 79.42(75.9-83.1) | 49.5(48.04-51.01) | 30.02(28.85-31.24) | 38.61(37.66-39.58) | 47.07(45.65-48.53) | 50.08(48.68-51.51) | 48.89(47.9-49.9) |
|  |  | 90-94 | 72.98(68.56-77.68) | 27.59(26.14-29.11) | 45.45(43.4-47.59) | 76.92(75.4-78.47) | 26.33(25.81-26.87) | 45.18(44.37-46.01) | 86.12(78.79-94.13) | 30.64(28.22-33.27) | 51.47(48.1-55.06) | 82.26(73.03-92.66) | 35.67(32.52-39.12) | 58.48(53.86-63.49) | 43.44(41.1-45.91) | 27.11(25.42-28.92) | 34.07(32.63-35.58) | 42.46(40.08-44.98) | 44.09(42.06-46.22) | 43.58(42.03-45.19) |
|  |  | ≥95 | 64.9(56.97-73.94) | 26.78(24.47-29.3) | 42.16(38.69-45.95) | 82.73(79.92-85.64) | 31.24(30.45-32.05) | 50.08(48.72-51.47) | 65.65(52.83-81.59) | 29.68(25.56-34.46) | 45.56(39.56-52.46) | 49.2(36.34-66.61) | 29.04(24.12-34.95) | 43.3(36.05-52) | 43.58(38.82-48.93) | 26.24(23.25-29.62) | 33.16(30.44-36.12) | 42.67(37.29-48.83) | 45.3(41.33-49.65) | 44.59(41.35-48.09) |
|  | Period | 1990-1994 | 0.99(0.97-1.02) | 1.06(1.04-1.09) | 1.01(0.99-1.03) | 1.03(1.02-1.04) | 1.05(1.03-1.06) | 1.03(1.02-1.04) | 0.98(0.95-1.01) | 1.13(1.08-1.19) | 1.01(0.98-1.03) | 1.03(0.99-1.06) | 1.16(1.11-1.2) | 1.06(1.03-1.09) | 0.99(0.98-1.01) | 1.05(1.03-1.08) | 1.02(1.01-1.03) | 0.99(0.97-1) | 1.06(1.05-1.08) | 1.02(1.01-1.03) |
|  |  | 1995-1999 | 0.96(0.94-0.98) | 1(0.97-1.02) | 0.97(0.96-0.99) | 1.02(1.01-1.03) | 1.03(1.01-1.04) | 1.02(1.01-1.02) | 0.95(0.92-0.97) | 1.02(0.98-1.06) | 0.96(0.94-0.98) | 0.96(0.93-0.99) | 1.02(0.98-1.05) | 0.98(0.95-1) | 0.99(0.98-1.01) | 1.03(1.01-1.05) | 1.01(1-1.02) | 1(0.99-1.02) | 1.04(1.03-1.06) | 1.02(1.01-1.03) |
|  |  | 2000-2004 | 1(1-1) | 1(1-1) | 1(1-1) | 1(1-1) | 1(1-1) | 1(1-1) | 1(1-1) | 1(1-1) | 1(1-1) | 1(1-1) | 1(1-1) | 1(1-1) | 1(1-1) | 1(1-1) | 1(1-1) | 1(1-1) | 1(1-1) | 1(1-1) |
|  |  | 2005-2009 | 0.94(0.92-0.96) | 0.87(0.85-0.89) | 0.92(0.9-0.93) | 0.97(0.96-0.98) | 0.94(0.93-0.96) | 0.97(0.96-0.98) | 0.93(0.91-0.95) | 0.84(0.81-0.87) | 0.91(0.89-0.93) | 0.91(0.89-0.94) | 0.8(0.78-0.83) | 0.88(0.86-0.9) | 0.98(0.97-0.99) | 0.93(0.91-0.95) | 0.96(0.95-0.97) | 0.97(0.96-0.98) | 0.94(0.92-0.95) | 0.96(0.95-0.97) |
|  |  | 2010-2014 | 0.82(0.81-0.84) | 0.7(0.68-0.71) | 0.78(0.77-0.8) | 0.93(0.92-0.94) | 0.9(0.88-0.91) | 0.93(0.92-0.94) | 0.81(0.79-0.84) | 0.66(0.63-0.69) | 0.78(0.76-0.8) | 0.74(0.72-0.76) | 0.55(0.53-0.57) | 0.67(0.66-0.69) | 0.93(0.92-0.94) | 0.84(0.83-0.86) | 0.89(0.88-0.9) | 0.95(0.94-0.96) | 0.89(0.88-0.91) | 0.92(0.91-0.93) |
|  |  | 2015-2019 | 0.76(0.74-0.78) | 0.64(0.62-0.65) | 0.72(0.71-0.74) | 0.91(0.9-0.93) | 0.87(0.85-0.88) | 0.92(0.9-0.93) | 0.77(0.74-0.79) | 0.6(0.57-0.63) | 0.73(0.71-0.75) | 0.64(0.61-0.66) | 0.45(0.43-0.47) | 0.57(0.55-0.59) | 0.92(0.9-0.93) | 0.83(0.82-0.85) | 0.88(0.86-0.89) | 0.93(0.92-0.94) | 0.88(0.87-0.89) | 0.9(0.9-0.91) |
|  | Cohort | 1895 | 1.07(0.65-1.74) | 1.85(1.38-2.49) | 1.28(0.95-1.74) | 0.78(0.68-0.89) | 1.19(1.11-1.28) | 0.86(0.78-0.95) | 1.19(0.56-2.51) | 1.81(1.11-2.93) | 1.3(0.81-2.09) | 1.68(0.59-4.82) | 3.27(1.78-6.02) | 2.08(1.12-3.87) | 1.07(0.69-1.66) | 1.63(1.08-2.46) | 1.33(0.98-1.81) | 1.1(0.69-1.74) | 1.08(0.79-1.48) | 1.09(0.84-1.4) |
|  |  | 1900 | 1.08(0.9-1.3) | 1.82(1.59-2.07) | 1.28(1.13-1.45) | 0.88(0.83-0.93) | 1.27(1.22-1.31) | 0.93(0.89-0.97) | 1.06(0.81-1.39) | 1.7(1.39-2.08) | 1.21(1-1.46) | 1.36(0.94-1.97) | 2.98(2.33-3.81) | 1.84(1.45-2.33) | 1.09(0.93-1.29) | 1.54(1.3-1.84) | 1.3(1.15-1.47) | 1.14(0.97-1.35) | 1.14(1-1.3) | 1.14(1.03-1.26) |
|  |  | 1905 | 1.05(0.97-1.15) | 1.79(1.66-1.92) | 1.24(1.17-1.32) | 0.97(0.94-1) | 1.31(1.28-1.35) | 0.98(0.96-1.01) | 0.98(0.88-1.1) | 1.65(1.48-1.84) | 1.13(1.03-1.24) | 1.18(1.02-1.37) | 2.84(2.51-3.21) | 1.67(1.5-1.86) | 1.09(1.01-1.17) | 1.48(1.36-1.62) | 1.26(1.19-1.34) | 1.16(1.07-1.25) | 1.16(1.09-1.23) | 1.15(1.1-1.21) |
|  |  | 1910 | 1.09(1.03-1.15) | 1.72(1.64-1.81) | 1.24(1.19-1.29) | 1.01(0.99-1.03) | 1.31(1.28-1.34) | 1(0.99-1.02) | 1(0.94-1.07) | 1.56(1.44-1.68) | 1.1(1.04-1.16) | 1.29(1.19-1.41) | 2.8(2.58-3.04) | 1.71(1.61-1.83) | 1.1(1.06-1.15) | 1.42(1.34-1.5) | 1.24(1.2-1.29) | 1.16(1.11-1.21) | 1.15(1.11-1.2) | 1.16(1.12-1.19) |
|  |  | 1915 | 1.17(1.13-1.22) | 1.79(1.71-1.86) | 1.31(1.27-1.35) | 1.06(1.05-1.08) | 1.33(1.3-1.35) | 1.05(1.03-1.07) | 1.09(1.04-1.15) | 1.6(1.5-1.7) | 1.16(1.11-1.22) | 1.39(1.31-1.47) | 2.75(2.58-2.93) | 1.75(1.67-1.84) | 1.14(1.1-1.17) | 1.4(1.34-1.46) | 1.25(1.22-1.28) | 1.16(1.12-1.19) | 1.17(1.13-1.2) | 1.16(1.14-1.19) |
|  |  | 1920 | 1.22(1.18-1.25) | 1.75(1.69-1.81) | 1.33(1.3-1.37) | 1.1(1.09-1.12) | 1.28(1.26-1.3) | 1.06(1.05-1.08) | 1.17(1.13-1.21) | 1.61(1.52-1.7) | 1.22(1.18-1.27) | 1.41(1.35-1.47) | 2.55(2.42-2.69) | 1.71(1.65-1.78) | 1.11(1.08-1.13) | 1.34(1.29-1.38) | 1.2(1.18-1.23) | 1.11(1.09-1.13) | 1.15(1.12-1.18) | 1.13(1.11-1.15) |
|  |  | 1925 | 1.2(1.17-1.23) | 1.61(1.56-1.67) | 1.29(1.26-1.32) | 1.12(1.1-1.13) | 1.21(1.19-1.23) | 1.08(1.07-1.09) | 1.11(1.08-1.15) | 1.44(1.37-1.51) | 1.14(1.11-1.18) | 1.37(1.33-1.42) | 2.29(2.19-2.4) | 1.62(1.57-1.67) | 1.07(1.05-1.09) | 1.25(1.21-1.29) | 1.15(1.13-1.17) | 1.06(1.05-1.08) | 1.13(1.1-1.15) | 1.1(1.08-1.11) |
|  |  | 1930 | 1.16(1.13-1.18) | 1.47(1.42-1.51) | 1.23(1.21-1.25) | 1.11(1.1-1.12) | 1.14(1.12-1.16) | 1.09(1.08-1.1) | 1.08(1.05-1.11) | 1.33(1.27-1.4) | 1.11(1.08-1.14) | 1.27(1.23-1.31) | 1.9(1.82-1.98) | 1.44(1.4-1.48) | 1.05(1.03-1.07) | 1.18(1.15-1.21) | 1.1(1.09-1.12) | 1.04(1.02-1.05) | 1.1(1.08-1.12) | 1.07(1.06-1.08) |
|  |  | 1935 | 1.11(1.08-1.13) | 1.29(1.26-1.33) | 1.15(1.13-1.17) | 1.07(1.06-1.08) | 1.07(1.05-1.08) | 1.06(1.05-1.07) | 1.05(1.02-1.07) | 1.22(1.16-1.27) | 1.07(1.04-1.1) | 1.17(1.13-1.2) | 1.52(1.46-1.59) | 1.26(1.22-1.29) | 1.05(1.04-1.07) | 1.11(1.09-1.14) | 1.07(1.06-1.09) | 1.04(1.02-1.05) | 1.07(1.05-1.08) | 1.05(1.04-1.06) |
|  |  | 1940 | 1.02(1-1.04) | 1.09(1.06-1.12) | 1.03(1.02-1.05) | 1.03(1.02-1.04) | 1(0.99-1.02) | 1.02(1.01-1.03) | 0.99(0.96-1.01) | 1.02(0.98-1.07) | 0.98(0.95-1.01) | 1.04(1.01-1.08) | 1.19(1.14-1.24) | 1.08(1.05-1.11) | 1.03(1.01-1.04) | 1.05(1.02-1.07) | 1.03(1.02-1.05) | 1.02(1-1.03) | 1.03(1.01-1.05) | 1.02(1.01-1.03) |
|  |  | 1945 | 1(1-1) | 1(1-1) | 1(1-1) | 1(1-1) | 1(1-1) | 1(1-1) | 1(1-1) | 1(1-1) | 1(1-1) | 1(1-1) | 1(1-1) | 1(1-1) | 1(1-1) | 1(1-1) | 1(1-1) | 1(1-1) | 1(1-1) | 1(1-1) |
|  |  | 1950 | 0.96(0.94-0.98) | 0.87(0.84-0.9) | 0.94(0.92-0.96) | 0.95(0.94-0.96) | 0.95(0.93-0.97) | 0.95(0.94-0.96) | 0.94(0.91-0.97) | 0.8(0.76-0.85) | 0.91(0.88-0.93) | 0.95(0.92-0.98) | 0.84(0.8-0.88) | 0.92(0.89-0.95) | 0.96(0.94-0.97) | 0.92(0.9-0.95) | 0.94(0.93-0.95) | 0.99(0.97-1) | 0.94(0.93-0.96) | 0.97(0.96-0.98) |
|  |  | 1955 | 0.89(0.87-0.91) | 0.74(0.72-0.77) | 0.85(0.83-0.87) | 0.89(0.88-0.9) | 0.91(0.89-0.93) | 0.89(0.88-0.91) | 0.87(0.85-0.9) | 0.66(0.62-0.7) | 0.83(0.8-0.85) | 0.83(0.81-0.86) | 0.64(0.6-0.68) | 0.78(0.75-0.81) | 0.94(0.93-0.96) | 0.88(0.86-0.9) | 0.92(0.9-0.93) | 0.99(0.97-1) | 0.9(0.89-0.92) | 0.95(0.94-0.96) |
|  |  | 1960 | 0.76(0.73-0.78) | 0.62(0.59-0.65) | 0.72(0.7-0.74) | 0.82(0.81-0.84) | 0.86(0.84-0.89) | 0.83(0.82-0.84) | 0.76(0.74-0.79) | 0.51(0.47-0.55) | 0.71(0.68-0.74) | 0.64(0.61-0.67) | 0.45(0.42-0.48) | 0.59(0.56-0.61) | 0.92(0.91-0.94) | 0.86(0.83-0.89) | 0.9(0.88-0.91) | 0.97(0.95-0.98) | 0.85(0.83-0.87) | 0.92(0.9-0.93) |
|  |  | 1965 | 0.72(0.7-0.75) | 0.57(0.54-0.6) | 0.68(0.66-0.7) | 0.75(0.74-0.77) | 0.81(0.79-0.84) | 0.76(0.75-0.78) | 0.74(0.7-0.77) | 0.44(0.4-0.49) | 0.68(0.64-0.71) | 0.59(0.56-0.63) | 0.37(0.34-0.41) | 0.53(0.5-0.56) | 0.92(0.9-0.94) | 0.84(0.81-0.87) | 0.88(0.86-0.9) | 0.95(0.93-0.96) | 0.8(0.78-0.82) | 0.88(0.87-0.9) |
|  |  | 1970 | 0.63(0.6-0.66) | 0.5(0.47-0.54) | 0.59(0.57-0.62) | 0.69(0.67-0.71) | 0.76(0.72-0.8) | 0.71(0.69-0.73) | 0.63(0.59-0.67) | 0.37(0.32-0.43) | 0.58(0.54-0.62) | 0.48(0.44-0.52) | 0.3(0.26-0.34) | 0.43(0.4-0.46) | 0.9(0.87-0.93) | 0.76(0.73-0.8) | 0.84(0.82-0.86) | 0.93(0.91-0.96) | 0.75(0.73-0.77) | 0.85(0.84-0.87) |
|  |  | 1975 | 0.55(0.51-0.59) | 0.47(0.42-0.52) | 0.52(0.49-0.56) | 0.71(0.68-0.74) | 0.75(0.69-0.81) | 0.72(0.69-0.76) | 0.54(0.49-0.6) | 0.31(0.25-0.39) | 0.49(0.44-0.55) | 0.38(0.34-0.42) | 0.24(0.2-0.29) | 0.34(0.3-0.38) | 0.89(0.86-0.93) | 0.76(0.71-0.81) | 0.83(0.8-0.86) | 0.94(0.91-0.97) | 0.7(0.67-0.73) | 0.83(0.81-0.85) |
|  |  | 1980 | 0.53(0.47-0.6) | 0.42(0.36-0.49) | 0.5(0.45-0.55) | 0.75(0.7-0.81) | 0.77(0.68-0.87) | 0.76(0.7-0.83) | 0.52(0.44-0.62) | 0.26(0.19-0.36) | 0.46(0.39-0.55) | 0.35(0.29-0.43) | 0.2(0.15-0.26) | 0.31(0.26-0.36) | 0.88(0.83-0.94) | 0.71(0.65-0.77) | 0.8(0.76-0.84) | 0.94(0.9-1) | 0.64(0.61-0.68) | 0.79(0.76-0.82) |
|  |  | 1985 | 0.53(0.44-0.64) | 0.37(0.3-0.46) | 0.47(0.4-0.55) | 0.8(0.71-0.91) | 0.77(0.64-0.93) | 0.81(0.7-0.92) | 0.52(0.39-0.69) | 0.22(0.14-0.35) | 0.44(0.33-0.57) | 0.35(0.26-0.47) | 0.16(0.11-0.24) | 0.28(0.21-0.36) | 0.86(0.79-0.95) | 0.66(0.59-0.73) | 0.76(0.71-0.82) | 0.94(0.87-1.02) | 0.59(0.55-0.63) | 0.74(0.7-0.78) |
|  |  | 1990 | 0.51(0.37-0.69) | 0.34(0.26-0.46) | 0.44(0.34-0.56) | 0.78(0.62-0.97) | 0.72(0.54-0.96) | 0.77(0.61-0.97) | 0.48(0.29-0.79) | 0.19(0.1-0.38) | 0.39(0.24-0.62) | 0.33(0.2-0.55) | 0.15(0.09-0.25) | 0.25(0.17-0.38) | 0.81(0.7-0.93) | 0.6(0.51-0.69) | 0.7(0.63-0.77) | 0.92(0.81-1.04) | 0.54(0.48-0.59) | 0.69(0.64-0.75) |
|  |  | 1995 | 0.48(0.28-0.81) | 0.31(0.19-0.5) | 0.41(0.27-0.62) | 0.68(0.44-1.06) | 0.67(0.4-1.14) | 0.69(0.44-1.07) | 0.43(0.18-1.07) | 0.17(0.05-0.58) | 0.35(0.15-0.81) | 0.32(0.13-0.75) | 0.13(0.05-0.31) | 0.23(0.11-0.47) | 0.79(0.63-1) | 0.52(0.41-0.66) | 0.64(0.54-0.76) | 0.92(0.76-1.12) | 0.5(0.42-0.59) | 0.67(0.59-0.76) |
| DALYs | Age | 20-24 | 8.68(7.44-10.13) | 12.33(10.78-14.11) | 9.85(8.76-11.06) | 2.99(2.66-3.37) | 1.24(1.13-1.37) | 2.13(1.91-2.39) | 9.34(7.64-11.42) | 12.36(9.42-16.23) | 8.96(7.49-10.72) | 15.14(11.87-19.31) | 27.82(22.12-35) | 18.07(14.93-21.86) | 5.62(5.2-6.08) | 11(10.2-11.86) | 7.96(7.55-8.38) | 4.23(4.06-4.41) | 9.39(9.09-9.7) | 6.42(6.23-6.61) |
|  |  | 25-29 | 14.24(12.71-15.97) | 19.12(17.23-21.21) | 15.69(14.37-17.12) | 6.2(5.73-6.71) | 2.28(2.13-2.44) | 4.28(3.97-4.62) | 13.65(11.73-15.89) | 16.68(13.54-20.54) | 12.77(11.14-14.63) | 23.03(19.19-27.64) | 37.47(31.34-44.79) | 25.98(22.47-30.03) | 10.4(9.81-11.02) | 17.83(16.79-18.93) | 13.67(13.13-14.23) | 7.93(7.69-8.19) | 19.87(19.41-20.34) | 13.05(12.78-13.33) |
|  |  | 30-34 | 37.02(34.42-39.81) | 28.45(26.18-30.92) | 31.83(29.92-33.86) | 16.79(16.01-17.61) | 4.03(3.84-4.23) | 10.53(10.04-11.04) | 39.13(35.76-42.83) | 24.96(21.28-29.26) | 29.75(27.24-32.49) | 62.41(55.77-69.83) | 54.92(47.78-63.13) | 54.56(49.42-60.24) | 23.03(22.08-24.02) | 25.23(23.98-26.55) | 23.9(23.15-24.67) | 19.57(19.14-20.01) | 32.59(31.97-33.22) | 25.19(24.78-25.61) |
|  |  | 35-39 | 92.22(87.92-96.74) | 45.45(42.54-48.56) | 68.48(65.58-71.52) | 45.2(43.89-46.55) | 8.66(8.38-8.96) | 27.21(26.41-28.04) | 98.07(92.58-103.89) | 39.4(34.93-44.45) | 67.13(63.3-71.18) | 152.42(141.71-163.94) | 76.19(68.16-85.18) | 112.87(105.38-120.9) | 55.34(53.68-57.05) | 42.78(41.02-44.61) | 49.23(48.05-50.45) | 52.55(51.74-53.38) | 61.4(60.46-62.35) | 56.13(55.44-56.83) |
|  |  | 40-44 | 239.5(231.82-247.44) | 86.08(81.85-90.53) | 164.36(159.42-169.44) | 125.77(123.45-128.13) | 19.82(19.37-20.29) | 73.38(71.98-74.8) | 265.2(255.22-275.57) | 70(64.01-76.56) | 168.51(161.93-175.35) | 390.93(372.13-410.69) | 141.16(130.04-153.23) | 269.81(257.32-282.9) | 128.49(125.58-131.47) | 84.46(81.66-87.37) | 107.47(105.46-109.52) | 146.89(145.23-148.56) | 120.58(119.08-122.09) | 133.98(132.71-135.26) |
|  |  | 45-49 | 436.74(425.82-447.95) | 144.43(138.84-150.25) | 293.74(286.85-300.79) | 272.31(268.76-275.91) | 40.9(40.23-41.58) | 157.26(155.14-159.41) | 516.73(501.92-531.97) | 119.94(112.18-128.25) | 320.47(310.97-330.26) | 640.67(615.9-666.45) | 222.18(208.4-236.88) | 437.54(421.35-454.35) | 248.67(244.22-253.2) | 147.99(143.99-152.1) | 200.57(197.58-203.61) | 279.73(277.21-282.27) | 211.05(208.91-213.21) | 246.46(244.58-248.35) |
|  |  | 50-54 | 722.55(707.3-738.12) | 226.37(219.05-233.95) | 478.04(468.6-487.67) | 479.05(473.99-484.16) | 76.14(75.16-77.14) | 277.49(274.47-280.54) | 868.39(847.47-889.82) | 190.21(180.19-200.77) | 529.48(516.36-542.93) | 1000.13(967.24-1034.14) | 332.5(315.45-350.46) | 674.26(653.18-696.02) | 426.32(419.76-432.97) | 233.3(227.77-238.96) | 332.95(328.65-337.3) | 513.21(509.27-517.18) | 352.99(349.89-356.13) | 435.54(432.69-438.41) |
|  |  | 55-59 | 990.19(971.24-1009.51) | 292.08(283.58-300.83) | 641.42(629.96-653.09) | 690.24(683.76-696.78) | 110.96(109.7-112.23) | 397.75(393.92-401.63) | 1208.83(1182.64-1235.61) | 253.88(242.22-266.09) | 722.88(706.85-739.26) | 1338.11(1298.14-1379.32) | 425.32(405.96-445.6) | 887.32(862.26-913.11) | 590.16(581.75-598.69) | 288.2(281.75-294.79) | 441.17(435.86-446.54) | 665.29(660.45-670.16) | 458.87(455.06-462.71) | 563.93(560.44-567.44) |
|  |  | 60-64 | 1170.15(1148.35-1192.35) | 351.01(341.31-360.98) | 753.7(740.67-766.96) | 843.22(835.66-850.85) | 138.86(137.38-140.35) | 483.18(478.73-487.66) | 1427.16(1396.85-1458.12) | 319.24(305.53-333.56) | 850.35(832.03-869.08) | 1582.05(1536.24-1629.22) | 531.83(509.26-555.4) | 1054.82(1026.23-1084.21) | 692.47(682.75-702.32) | 308.27(301.39-315.3) | 498.98(493.05-504.98) | 772.59(766.98-778.24) | 508.54(504.3-512.82) | 641.94(637.96-645.95) |
|  |  | 65-69 | 1268.78(1244.11-1293.94) | 395.18(384.03-406.65) | 816.03(801.39-830.94) | 922.09(913.67-930.58) | 159.58(157.9-161.28) | 525.03(520.13-529.97) | 1483.22(1449.96-1517.24) | 367.26(351.32-383.92) | 887.24(867.26-907.69) | 1784.2(1730.39-1839.69) | 607.51(581.1-635.13) | 1183.31(1150.06-1217.52) | 733.25(722.41-744.24) | 339.83(332.02-347.82) | 531.86(525.24-538.55) | 808.11(801.84-814.43) | 533.51(528.81-538.26) | 671.12(666.69-675.58) |
|  |  | 70-74 | 1314.73(1286.51-1343.57) | 421.68(409.19-434.56) | 841.13(824.71-857.88) | 924.85(915.73-934.05) | 178.2(176.27-180.16) | 525.57(520.33-530.87) | 1544.49(1506.35-1583.6) | 398.69(380.81-417.41) | 915.03(892.7-937.91) | 1906.39(1842.97-1972) | 641.41(612.07-672.15) | 1247.46(1209.36-1286.76) | 700.69(688.85-712.74) | 379.82(370.46-389.4) | 533.34(525.93-540.86) | 788.55(781.49-795.67) | 577.46(571.86-583.11) | 681.53(676.44-686.65) |
|  |  | 75-79 | 1200.15(1167.3-1233.93) | 386.1(372.4-400.31) | 753.91(735.51-772.78) | 861.36(850.73-872.13) | 195.99(193.52-198.48) | 492.64(486.6-498.76) | 1422.98(1378.44-1468.96) | 379.12(359.08-400.27) | 828.09(803.05-853.91) | 1738.59(1664.98-1815.44) | 571.34(540.26-604.2) | 1113.24(1070.94-1157.21) | 643.4(628.96-658.18) | 371.03(359.45-382.98) | 498.27(489.25-507.46) | 650.01(641.91-658.21) | 499.18(492.7-505.75) | 571.86(566.02-577.75) |
|  |  | 80-84 | 1009.69(973.59-1047.13) | 322.9(309.31-337.09) | 616.69(597.69-636.3) | 763.58(752-775.34) | 200.27(197.52-203.06) | 437.04(430.68-443.48) | 1229.46(1179.7-1281.32) | 328.95(308.88-350.34) | 692.27(666.19-719.36) | 1435.45(1354.75-1520.94) | 467.47(437.05-500) | 900.28(856.57-946.22) | 526.67(510.12-543.77) | 299.67(287.32-312.55) | 402.41(392.43-412.65) | 532.61(523.04-542.35) | 476.89(468.79-485.12) | 503.36(496.3-510.52) |
|  |  | 85-89 | 859.26(812.8-908.37) | 265.11(250.28-280.82) | 498.77(476.62-521.95) | 681.17(666.97-695.67) | 208.84(205.57-212.16) | 394.9(387.52-402.43) | 1100.01(1032.99-1171.37) | 278.77(256.6-302.86) | 577.39(546.23-610.33) | 1209.64(1102.76-1326.87) | 361.03(328.34-396.98) | 712.56(660.54-768.68) | 448.14(425.39-472.1) | 272.3(255.87-289.8) | 349.76(336.3-363.76) | 426.86(413.84-440.28) | 453.02(441.61-464.73) | 442.62(432.81-452.66) |
|  |  | 90-94 | 511.33(451.92-578.54) | 191.5(173.92-210.85) | 316.84(290.08-346.07) | 541.48(521.99-561.7) | 184.91(181.09-188.82) | 317.6(308.37-327.11) | 603.59(517.74-703.66) | 211.37(183.9-242.95) | 357.65(319.28-400.63) | 578.88(452.53-740.49) | 245.91(206.28-293.15) | 407.52(344.63-481.89) | 304.54(271.72-341.33) | 190.58(168.78-215.19) | 239.09(220.41-259.35) | 297.69(277.88-318.91) | 308.32(292.93-324.52) | 304.89(290.86-319.6) |
|  |  | ≥95 | 339.78(250.34-461.17) | 139.97(114.92-170.47) | 220.72(181.3-268.7) | 436.08(403.83-470.91) | 163.88(158.49-169.46) | 263.34(249.3-278.16) | 346.89(223.06-539.47) | 155.34(113.68-212.25) | 239.81(180.74-318.2) | 257.64(122.99-539.72) | 152.53(99.54-233.74) | 228.27(146.7-355.2) | 229.06(173.02-303.25) | 138.5(105.55-181.72) | 174.54(144.29-211.13) | 231.12(191.87-278.4) | 240.97(214.24-271.03) | 238.26(212.45-267.22) |
|  | Period | 1990-1994 | 0.98(0.96-1.01) | 1.04(1.01-1.07) | 1(0.98-1.02) | 1.03(1.02-1.04) | 1.04(1.03-1.05) | 1.03(1.02-1.04) | 0.97(0.94-1) | 1.09(1.04-1.13) | 0.99(0.96-1.01) | 1.01(0.97-1.05) | 1.12(1.07-1.17) | 1.04(1.01-1.07) | 0.99(0.97-1) | 1.06(1.03-1.08) | 1.02(1-1.03) | 0.98(0.97-0.99) | 1.06(1.05-1.07) | 1.02(1.01-1.03) |
|  |  | 1995-1999 | 0.96(0.94-0.98) | 0.99(0.97-1.02) | 0.97(0.95-0.98) | 1.02(1.01-1.03) | 1.02(1.01-1.03) | 1.02(1.01-1.02) | 0.94(0.92-0.96) | 1(0.96-1.04) | 0.95(0.93-0.97) | 0.95(0.92-0.98) | 1.01(0.97-1.05) | 0.97(0.94-1) | 0.99(0.98-1.01) | 1.04(1.02-1.06) | 1.01(1-1.03) | 1(1-1.01) | 1.04(1.04-1.05) | 1.02(1.02-1.03) |
|  |  | 2000-2004 | 1(1-1) | 1(1-1) | 1(1-1) | 1(1-1) | 1(1-1) | 1(1-1) | 1(1-1) | 1(1-1) | 1(1-1) | 1(1-1) | 1(1-1) | 1(1-1) | 1(1-1) | 1(1-1) | 1(1-1) | 1(1-1) | 1(1-1) | 1(1-1) |
|  |  | 2005-2009 | 0.94(0.93-0.96) | 0.86(0.84-0.88) | 0.92(0.9-0.93) | 0.97(0.96-0.98) | 0.95(0.94-0.96) | 0.97(0.96-0.98) | 0.94(0.92-0.96) | 0.84(0.81-0.87) | 0.92(0.9-0.93) | 0.91(0.89-0.94) | 0.79(0.76-0.82) | 0.87(0.85-0.9) | 0.98(0.97-1) | 0.92(0.9-0.94) | 0.95(0.94-0.97) | 0.97(0.96-0.98) | 0.93(0.92-0.94) | 0.95(0.95-0.96) |
|  |  | 2010-2014 | 0.82(0.8-0.84) | 0.69(0.67-0.71) | 0.78(0.76-0.79) | 0.93(0.92-0.94) | 0.89(0.89-0.9) | 0.93(0.92-0.94) | 0.81(0.79-0.83) | 0.65(0.62-0.67) | 0.77(0.75-0.79) | 0.73(0.7-0.76) | 0.54(0.52-0.57) | 0.67(0.64-0.69) | 0.93(0.91-0.95) | 0.85(0.83-0.87) | 0.89(0.88-0.9) | 0.94(0.94-0.95) | 0.89(0.88-0.9) | 0.92(0.91-0.93) |
|  |  | 2015-2019 | 0.75(0.73-0.77) | 0.63(0.61-0.65) | 0.71(0.7-0.73) | 0.91(0.9-0.93) | 0.86(0.85-0.87) | 0.92(0.91-0.93) | 0.76(0.73-0.78) | 0.58(0.56-0.61) | 0.72(0.7-0.74) | 0.63(0.6-0.66) | 0.44(0.42-0.46) | 0.56(0.54-0.58) | 0.91(0.89-0.93) | 0.84(0.82-0.86) | 0.87(0.86-0.89) | 0.93(0.91-0.94) | 0.88(0.87-0.89) | 0.9(0.89-0.91) |
|  | Cohort | 1895 | 1.1(0.34-3.53) | 1.92(0.98-3.79) | 1.33(0.66-2.68) | 0.79(0.58-1.08) | 1.21(1.08-1.36) | 0.87(0.71-1.07) | 1.23(0.27-5.68) | 1.9(0.67-5.42) | 1.35(0.51-3.53) | 1.76(0.13-22.9) | 3.43(0.82-14.43) | 2.16(0.48-9.76) | 1.1(0.38-3.21) | 1.65(0.64-4.21) | 1.36(0.68-2.69) | 1.11(0.58-2.12) | 1.09(0.73-1.64) | 1.1(0.74-1.64) |
|  |  | 1900 | 1.1(0.74-1.63) | 1.86(1.43-2.42) | 1.3(1-1.69) | 0.88(0.79-0.99) | 1.27(1.21-1.34) | 0.93(0.86-1.01) | 1.09(0.67-1.76) | 1.77(1.2-2.6) | 1.24(0.89-1.74) | 1.38(0.62-3.08) | 3.07(1.84-5.14) | 1.87(1.12-3.14) | 1.11(0.78-1.58) | 1.54(1.08-2.19) | 1.31(1.02-1.67) | 1.15(0.94-1.42) | 1.15(0.99-1.34) | 1.15(1-1.32) |
|  |  | 1905 | 1.07(0.91-1.25) | 1.82(1.6-2.07) | 1.26(1.12-1.41) | 0.98(0.92-1.03) | 1.32(1.29-1.36) | 0.99(0.95-1.03) | 1(0.84-1.19) | 1.71(1.43-2.04) | 1.15(1-1.33) | 1.2(0.9-1.59) | 2.91(2.32-3.65) | 1.69(1.37-2.08) | 1.09(0.95-1.26) | 1.48(1.27-1.73) | 1.27(1.14-1.4) | 1.17(1.07-1.27) | 1.16(1.09-1.24) | 1.16(1.1-1.23) |
|  |  | 1910 | 1.1(1.02-1.2) | 1.74(1.61-1.89) | 1.25(1.17-1.33) | 1.01(0.98-1.04) | 1.31(1.28-1.34) | 1(0.98-1.03) | 1.02(0.93-1.12) | 1.59(1.43-1.78) | 1.12(1.03-1.21) | 1.32(1.14-1.52) | 2.86(2.51-3.25) | 1.74(1.56-1.94) | 1.11(1.03-1.19) | 1.42(1.3-1.55) | 1.25(1.18-1.32) | 1.17(1.12-1.22) | 1.16(1.11-1.2) | 1.16(1.13-1.2) |
|  |  | 1915 | 1.19(1.13-1.25) | 1.8(1.71-1.91) | 1.33(1.27-1.38) | 1.06(1.04-1.09) | 1.32(1.3-1.34) | 1.05(1.03-1.07) | 1.12(1.05-1.19) | 1.63(1.5-1.77) | 1.19(1.12-1.25) | 1.41(1.3-1.53) | 2.8(2.57-3.05) | 1.78(1.66-1.91) | 1.14(1.09-1.2) | 1.4(1.32-1.48) | 1.25(1.21-1.3) | 1.16(1.13-1.19) | 1.17(1.14-1.2) | 1.17(1.15-1.19) |
|  |  | 1920 | 1.23(1.19-1.28) | 1.76(1.68-1.83) | 1.34(1.3-1.39) | 1.1(1.08-1.12) | 1.27(1.25-1.29) | 1.06(1.05-1.08) | 1.19(1.14-1.24) | 1.63(1.53-1.74) | 1.24(1.19-1.29) | 1.43(1.35-1.51) | 2.59(2.42-2.76) | 1.73(1.65-1.82) | 1.11(1.08-1.14) | 1.33(1.28-1.39) | 1.2(1.17-1.23) | 1.11(1.09-1.13) | 1.15(1.13-1.17) | 1.13(1.12-1.15) |
|  |  | 1925 | 1.21(1.17-1.24) | 1.62(1.56-1.68) | 1.29(1.26-1.33) | 1.11(1.1-1.13) | 1.21(1.19-1.22) | 1.08(1.06-1.09) | 1.12(1.08-1.16) | 1.44(1.37-1.52) | 1.15(1.11-1.19) | 1.38(1.32-1.45) | 2.31(2.19-2.44) | 1.63(1.57-1.7) | 1.07(1.05-1.1) | 1.25(1.2-1.29) | 1.15(1.12-1.17) | 1.07(1.05-1.08) | 1.13(1.11-1.14) | 1.1(1.09-1.11) |
|  |  | 1930 | 1.16(1.13-1.19) | 1.46(1.42-1.51) | 1.23(1.2-1.26) | 1.11(1.1-1.12) | 1.14(1.13-1.15) | 1.09(1.08-1.1) | 1.08(1.05-1.11) | 1.32(1.26-1.39) | 1.11(1.08-1.14) | 1.27(1.22-1.32) | 1.9(1.81-2) | 1.44(1.39-1.49) | 1.05(1.03-1.07) | 1.18(1.14-1.21) | 1.11(1.09-1.12) | 1.04(1.03-1.05) | 1.1(1.09-1.12) | 1.07(1.06-1.08) |
|  |  | 1935 | 1.11(1.08-1.13) | 1.29(1.25-1.33) | 1.15(1.13-1.17) | 1.07(1.06-1.08) | 1.06(1.05-1.07) | 1.06(1.05-1.07) | 1.04(1.02-1.07) | 1.21(1.15-1.27) | 1.07(1.04-1.1) | 1.17(1.13-1.21) | 1.52(1.45-1.6) | 1.26(1.22-1.3) | 1.05(1.03-1.07) | 1.12(1.09-1.14) | 1.07(1.06-1.09) | 1.03(1.02-1.04) | 1.07(1.06-1.08) | 1.05(1.04-1.06) |
|  |  | 1940 | 1.02(1-1.04) | 1.08(1.05-1.12) | 1.03(1.01-1.05) | 1.03(1.02-1.04) | 1(0.99-1.01) | 1.02(1.01-1.03) | 0.99(0.96-1.01) | 1.01(0.97-1.06) | 0.98(0.96-1.01) | 1.04(1.01-1.08) | 1.18(1.13-1.24) | 1.08(1.05-1.11) | 1.03(1.01-1.04) | 1.05(1.02-1.08) | 1.03(1.02-1.05) | 1.01(1.01-1.02) | 1.03(1.02-1.04) | 1.02(1.01-1.03) |
|  |  | 1945 | 1(1-1) | 1(1-1) | 1(1-1) | 1(1-1) | 1(1-1) | 1(1-1) | 1(1-1) | 1(1-1) | 1(1-1) | 1(1-1) | 1(1-1) | 1(1-1) | 1(1-1) | 1(1-1) | 1(1-1) | 1(1-1) | 1(1-1) | 1(1-1) |
|  |  | 1950 | 0.97(0.95-0.99) | 0.88(0.85-0.9) | 0.94(0.92-0.96) | 0.95(0.94-0.96) | 0.95(0.94-0.96) | 0.95(0.94-0.96) | 0.94(0.92-0.96) | 0.81(0.77-0.85) | 0.91(0.89-0.93) | 0.96(0.93-0.99) | 0.85(0.81-0.9) | 0.93(0.9-0.96) | 0.96(0.94-0.97) | 0.92(0.9-0.95) | 0.94(0.93-0.95) | 0.99(0.98-1) | 0.94(0.94-0.95) | 0.97(0.96-0.97) |
|  |  | 1955 | 0.9(0.88-0.92) | 0.75(0.72-0.78) | 0.86(0.84-0.88) | 0.9(0.89-0.91) | 0.92(0.9-0.93) | 0.9(0.89-0.91) | 0.88(0.85-0.9) | 0.67(0.63-0.7) | 0.83(0.81-0.85) | 0.84(0.81-0.87) | 0.65(0.61-0.69) | 0.79(0.76-0.81) | 0.94(0.93-0.96) | 0.88(0.86-0.9) | 0.92(0.9-0.93) | 0.99(0.98-0.99) | 0.9(0.9-0.91) | 0.95(0.94-0.96) |
|  |  | 1960 | 0.76(0.74-0.78) | 0.62(0.6-0.65) | 0.72(0.7-0.74) | 0.82(0.81-0.84) | 0.87(0.86-0.88) | 0.83(0.82-0.84) | 0.77(0.75-0.79) | 0.52(0.48-0.55) | 0.72(0.7-0.74) | 0.64(0.62-0.67) | 0.45(0.42-0.49) | 0.59(0.57-0.61) | 0.92(0.91-0.94) | 0.86(0.83-0.88) | 0.89(0.88-0.91) | 0.97(0.96-0.97) | 0.85(0.84-0.86) | 0.91(0.91-0.92) |
|  |  | 1965 | 0.73(0.71-0.75) | 0.58(0.55-0.61) | 0.69(0.67-0.71) | 0.76(0.74-0.77) | 0.82(0.8-0.84) | 0.77(0.75-0.78) | 0.74(0.72-0.77) | 0.46(0.42-0.5) | 0.69(0.66-0.71) | 0.6(0.57-0.63) | 0.38(0.35-0.42) | 0.54(0.51-0.56) | 0.92(0.9-0.94) | 0.83(0.81-0.86) | 0.88(0.87-0.9) | 0.95(0.94-0.96) | 0.8(0.79-0.81) | 0.88(0.87-0.89) |
|  |  | 1970 | 0.63(0.61-0.66) | 0.51(0.48-0.55) | 0.6(0.58-0.62) | 0.7(0.68-0.72) | 0.77(0.74-0.79) | 0.71(0.7-0.73) | 0.64(0.61-0.67) | 0.38(0.34-0.43) | 0.59(0.56-0.62) | 0.49(0.46-0.52) | 0.31(0.27-0.34) | 0.44(0.41-0.46) | 0.91(0.88-0.93) | 0.76(0.73-0.79) | 0.84(0.82-0.86) | 0.93(0.92-0.95) | 0.75(0.74-0.76) | 0.85(0.84-0.86) |
|  |  | 1975 | 0.56(0.52-0.59) | 0.47(0.44-0.51) | 0.53(0.5-0.56) | 0.71(0.69-0.74) | 0.76(0.73-0.79) | 0.73(0.7-0.75) | 0.55(0.51-0.59) | 0.32(0.27-0.38) | 0.5(0.47-0.54) | 0.39(0.35-0.42) | 0.25(0.21-0.29) | 0.35(0.32-0.38) | 0.9(0.87-0.93) | 0.75(0.72-0.79) | 0.83(0.81-0.86) | 0.94(0.93-0.96) | 0.7(0.69-0.71) | 0.83(0.81-0.84) |
|  |  | 1980 | 0.54(0.49-0.59) | 0.42(0.38-0.47) | 0.51(0.47-0.55) | 0.76(0.72-0.8) | 0.78(0.73-0.83) | 0.77(0.73-0.81) | 0.53(0.47-0.6) | 0.27(0.22-0.34) | 0.47(0.42-0.53) | 0.36(0.31-0.42) | 0.2(0.17-0.25) | 0.31(0.27-0.36) | 0.89(0.85-0.93) | 0.7(0.66-0.75) | 0.8(0.77-0.83) | 0.94(0.92-0.97) | 0.64(0.62-0.65) | 0.79(0.77-0.8) |
|  |  | 1985 | 0.54(0.47-0.62) | 0.38(0.33-0.44) | 0.48(0.43-0.54) | 0.81(0.74-0.88) | 0.78(0.72-0.85) | 0.81(0.74-0.88) | 0.53(0.44-0.63) | 0.23(0.17-0.31) | 0.45(0.38-0.53) | 0.36(0.28-0.45) | 0.17(0.13-0.22) | 0.28(0.23-0.35) | 0.87(0.81-0.93) | 0.65(0.6-0.7) | 0.76(0.72-0.8) | 0.94(0.91-0.97) | 0.59(0.57-0.6) | 0.74(0.72-0.76) |
|  |  | 1990 | 0.52(0.42-0.64) | 0.35(0.29-0.42) | 0.45(0.38-0.53) | 0.78(0.67-0.91) | 0.73(0.64-0.84) | 0.77(0.67-0.89) | 0.49(0.36-0.66) | 0.21(0.13-0.32) | 0.4(0.3-0.53) | 0.34(0.24-0.49) | 0.15(0.1-0.22) | 0.26(0.19-0.35) | 0.82(0.74-0.91) | 0.59(0.53-0.65) | 0.7(0.65-0.75) | 0.92(0.87-0.97) | 0.53(0.51-0.55) | 0.69(0.67-0.72) |
|  |  | 1995 | 0.49(0.34-0.7) | 0.32(0.23-0.44) | 0.42(0.32-0.55) | 0.69(0.52-0.92) | 0.69(0.54-0.86) | 0.69(0.53-0.9) | 0.45(0.26-0.76) | 0.19(0.09-0.39) | 0.37(0.23-0.59) | 0.33(0.18-0.59) | 0.13(0.07-0.24) | 0.24(0.15-0.39) | 0.81(0.68-0.95) | 0.51(0.44-0.6) | 0.64(0.57-0.72) | 0.92(0.85-1) | 0.49(0.46-0.52) | 0.67(0.63-0.71) |
